# Effects of leaf herbivory and autumn seasonality on plant secondary metabolites: A meta‐analysis

**DOI:** 10.1002/ece3.10912

**Published:** 2024-02-13

**Authors:** Lota Skovmand, Rose E. O'Dea, Keri A. Greig, Katherine R. Amato, Andrew P. Hendry

**Affiliations:** ^1^ Redpath Museum & Department of Biology McGill University Montreal Quebec Canada; ^2^ School of Agriculture, Food, and Ecosystem Sciences University of Melbourne Melbourne Victoria Australia; ^3^ Department of Integrative Biology University of Texas at Austin Austin Texas USA; ^4^ Department of Anthropology Northwestern University Evanston Illinois USA

**Keywords:** autumn seasonality, flavonoids, herbivory, phenolics, plant chemical defense, plant secondary metabolites

## Abstract

Plant secondary metabolites (PSMs) are produced by plants to overcome environmental challenges, both biotic and abiotic. We were interested in characterizing how autumn seasonality in temperate and subtropical climates affects overall PSM production in comparison to herbivory. Herbivory is commonly measured between spring to summer when plants have high resource availability and prioritize growth and reproduction. However, autumn seasonality also challenges plants as they cope with limited resources and prepare survival for winter. This suggests a potential gap in our understanding of how herbivory affects PSM production in autumn compared to spring/summer. Using meta‐analysis, we recorded overall production of 22 different PSM subgroups from 58 published papers to calculate effect sizes from herbivory studies (absence to presence) and temperate to subtropical seasonal studies (summer to autumn), while considering other variables (e.g., plant type, increase in time since herbivory, temperature, and precipitation). We also compared production of five phenolic PSM subgroups – hydroxybenzoic acids, flavan‐3‐ols, flavonols, hydrolysable tannins, and condensed tannins. We wanted to detect a shared response across all PSMs and found that herbivory increased overall PSM production in herbaceous plants. Herbivory was also found to have a positive effect on individual PSM subgroups, such as flavonol production, while autumn seasonality was found to have a positive effect on flavan‐3‐ol and condensed tannin production. We discuss how these responses might stem from plants producing some PSMs constitutively, whereas others are induced only after herbivory, and how plants produce metabolites with higher costs only during seasons when other resources for growth and reproduction are less available, while other phenolic PSM subgroups serve more than one function for plants and such functions can be season dependent. The outcome of our meta‐analysis is that autumn seasonality changes some PSM production differently from herbivory, and we see value in further investigating seasonality–herbivory interactions with plant chemical defense.

## Graphical abstract

Plant secondary metabolites (PSMs) are important plant responses to environmental stressors. In this meta‐analysis, we test how two stressors – herbivory and non‐growing seasonality – affect overall PSM production.

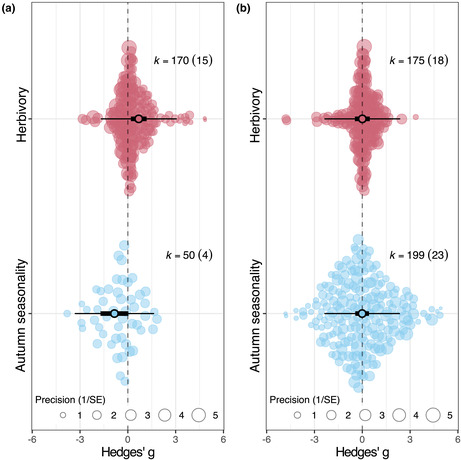

## 1 INTRODUCTION

Plants produce a broad array of diverse chemical compounds that can be upregulated or downregulated as adaptive responses to environmental challenges (Bourgaud et al., 2001; Pichersky & Gang, 2000; Wink, 2003). The production of these plant secondary metabolites (PSMs) is a multidimensional trait that serves various functions, such as deterring herbivores (Bennett & Wallsgrove, 1994), attracting predators and parasitoids of herbivores (Poelman et al., 2012), attracting pollinators (Pichersky & Gershenzon, 2002), and protecting the plant from other environmental stressors, including cold temperatures, water stress, and drought (Akula & Ravishankar, 2011; Ashraf et al., 2018). Any changes in PSM production also depend on structure and functionality of each chemical subgroup (Solar et al., 2006), and these subgroups do not exhibit one exclusive purpose but rather perform several functions in plants (Table 1).

**TABLE 1:** Description of the five largest phenolic plant secondary metabolism subgroups found in the dataset, highlighting structural differences and functional similarities.

	Structure example	Description of phenolic PSM subgroups and their relationships
A	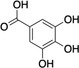	Hydroxybenzoic acids (HAs) are simple phenolic acids, such as gallic acids in this study. These metabolites are the primary building blocks of HTs (D) and derived via the shikimate biosynthetic pathway (Salminen & Karonen, 2011) HAs have pro‐oxidant activity and produce free radicals that are cytotoxic in guts (Salminen & Karonen, 2011). Like other phenolic PSM subgroups, HAs are induced by plants following herbivory (Mandal et al., 2010; Metlen et al., 2009) Example: Gallic acid
B	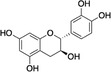	Flavan‐3‐ols are catechin monomers and dimers classified as flavonoids. These metabolites are the primary building blocks of CTs (E). Flavan‐3‐ols are induced by plants for anti‐herbivory defense (Thelen et al., 2005), yet also reduced following attacks in certain cases (Moctezuma et al., 2014) Production of flavan‐3‐ol varies between seasons for different plants, with some catechins increasing from spring to autumn (Liu et al., 2015). These metabolites therefore likely play a seasonal role for plants, such as in litter decomposition and nutrient cycling (Kraus et al., 2003) Example: Catechin
C	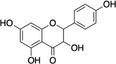	Flavonols are flavonoids with quercetin, kaempferol, and myricetin being most common in the paper. Flavonols are produced by plants to cope with abiotic stressors (Defossez et al., 2021) and herbivore attacks (Chin et al., 2018; Mierziak et al., 2014) Flavonols also have dual fluorescence and contribute to plant UV protection and flower color, making them critical for plant adaptation to climate stressors (Laoué et al., 2022; Middleton & Teramura, 1993; Smith & Markham, 1998) Example: Kaempferol
D	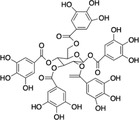	Hydrolysable tannins (HTs) are carbohydrate centers esterified with gallic acid (A) and other derivatives. HTs include gallotannin (GTs) and ellagitannin (ETs). The production of these metabolites cost 0.270 ATP equivalents/g per one HT (Lewis & Yamamoto, 1989), which is notably less than condensed tannin (E) When consumed by herbivores, HTs are hydrolyzed by weak acids and produce free radicals (Barbehenn & Peter Constabel, 2011), similarly to hydroxybenzoic acids (A). Most HTs are absorbed better in basic conditions than other metabolites and effectively disrupt the alkaline gut of insects (Tuominen & Sundman, 2013) Still, the functionality differs among HT members: while ETs oxidize efficiently in insect guts, GTs are less oxidatively active and function rather as biological antioxidants or feeding deterrents similarly to CTs (E) (Salminen & Karonen, 2011) Example: Gallotannin
E	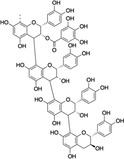	Condensed tannins (CTs) are flavan‐3‐ol oligomers (B) of catechin and other derivatives. These metabolites are complex polyphenols and metabolically costly for plants to produce (approximately 0.395 ATP equivalents/g per one CT) (Lewis & Yamamoto, 1989) CTs are well studied in plants for their anti‐herbivory defenses (Kraus et al., 2003). Like some HTs (D), CTs oxidate in the gut and impede digestion (Clausen et al., 1992; Salminen & Karonen, 2011). However, some evidence suggests CTs to function more as herbivore deterrents due to the bitterness from tannins binding to salivary proteins (Barbehenn & Peter Constabel, 2011) CTs also have antioxidant properties like flavonols (C) (Salminen, 2018; Salminen & Karonen, 2011), protect plants against oxidative stress (Gourlay & Constabel, 2019), and play a role in plant–litter–soil interactions (Kraus et al., 2003) Example: Proanthocyanidin

A rich literature of meta‐analyses documents the effects of herbivory on flavonoids, terpenoids, alkaloids, and other individual PSMs across plant species, including deciduous and evergreen trees (Nykänen & Koricheva, 2004; Ojha et al., 2022), woody and herbaceous plants (Barton et al., 2019; Barton & Koricheva, 2010), and field and greenhouse studies (Ojha et al., 2022). The meta‐analyses also compare effects of plant and leaf ontogeny (Barton et al., 2019; Barton & Koricheva, 2010; Nykänen & Koricheva, 2004), other abiotic factors, such as drought (Koricheva et al., 1998), and seasonal effects between early to late growing months (Nykänen & Koricheva, 2004). Indeed, most plant studies on herbivory are conducted between early to late growing season (i.e., from spring to late summer). To the best of our knowledge, no cumulative meta‐analysis has systematically compared the effects of herbivory to early autumn months on overall PSM production (Box 1) or among subgroup PSMs, such as condensed tannins, hydrolyzable tannins, and their derivatives, in plants. It is ecologically meaningful to understand how herbivory and autumn seasonality influence overall PSM production, as warmer autumn months are becoming more prevalent, herbivory during the late growing season is extending into autumn months, and plants are applying new chemical defense strategies in such changing environments.

BOX 1 **Definitions used in this paper**

1. *Autumn seasonality*: early non‐growing season in temperate and subtropical regions. Months considered in this paper were September–October in the northern hemispheres and April–May in the Southern hemisphere. Tropical regions were not included in the meta‐analysis.
2. *Constitutive response*: resistance that is always expressed in the plant (independent of damage).
3. *Induced response*: resistance that is expressed only after a plant is damaged.
4. *Metabolic subgroups*: chemical classification of PSMs used for the meta‐analysis (Table S2). Subgroups were determined based on main structural similarities and various literature (Ferreira et al., 1999; Twaij & Hasan, 2022) as the measurement specification and classification differed between studies and years.
5. *Overall PSM production*: all plant secondary metabolite effect sizes across all chemical subgroups collected for the meta‐analysis.
6. *Plant type*: type of plant based on structural tissue. Plants were denoted as woody (i.e., plants with woody structural tissue) or herbaceous (i.e., plants without woody structural tissue).
7. *Study type*: type of dataset used for the meta‐analysis. Study type was considered ‘herbivory’ if absence versus presence of herbivory was the focus of measurement or ‘autumn seasonality’ if summer to early autumn months was the focus of measurement.

### 1.1 Role of herbivory and autumn seasonality on PSMs

Seasonal variation in the production of PSMs is documented throughout the plant kingdom. In temperate and some subtropical regions, the growing season (i.e., spring and summer) has higher resource availability for plant growth while the non‐growing season (i.e., autumn and winter) has limited resource availability where plants must acclimate to abiotic changes (Fürtauer et al., 2019; Srivastava et al., 2021; Yang et al., 2018). PSM production across seasons is also suggested to be species dependent (Lindroth et al., 1987; Wiesneth et al., 2018). For example, *Betula pubescens* Ehrh and *Barbarea vulgaris* W. T. Aiton ssp. *arcuate* were found to increase PSM production from spring until winter (Agerbirk et al., 2001; Salminen et al., 2001), while *Populus grandidentata* Michx., declined in PSM production from late spring until the end of growing season (Lindroth et al., 1987). Plant type (Box 1) also factor into seasonal differences, as woody plants generally rely on compensatory growth with a constitutive response of PSMs throughout one season, while herbaceous plants have limited reserves (Boeckler et al., 2013; Feeny, 1976; Haukioja & Koricheva, 2000).

PSM production in response to herbivory has also been widely studied, specifically the upregulation of certain PSMs after herbivore attacks, also known as induced defensive response (Box 1), which allow plants to produce PSMs in a less costly manner than constitutive responses (McKey, 1974, 1979; Rhoades, 1979; Verma & Shukla, 2015). The plant pays the cost of defense only when required following herbivory and diminish over time, thereby giving a fitness advantage (Haukioja, 1980; Herms & Mattson, 1992; Karban & Myers, 1989; Kogan & Paxton, 1983; Tuomi et al., 1990; Zangerl & Bazzaz, 1992). Induced responses after herbivore attack can lower the chance of subsequent attack (Agrawal, 1998), increase the negative consequences of feeding to the attacker (Green & Ryan, 1972), and attract natural predators of herbivores (Unsicker et al., 2009). However, induced responses to herbivory differ with which species, specialization, or feeding guild of herbivore has attacked the plant (Klimm et al., 2020). Therefore, the upregulation of PSM production only occurs in the specific chemical subgroups, which targets the attacker (Baldwin, 1998). Also, not all PSMs exhibit inducibility and can be expressed constitutively depending on the frequency of stressors (Kessler, 2015).

Yet autumn's interactions with herbivory are generally understudied (Gallinat et al., 2015; Seifert et al., 2021; Wolda, 1988; Zani et al., 2020). Such interactions are relevant because insects can be active during the non‐growing season (i.e., autumn and winter), such some leafhoppers (Stinson & Brown, 1983), sawflies (Kause et al., 2001), and larvae of leaf‐mining moths (Connor et al., 1994), which re‐appear with a second generation during late summer and/or consume leaves until senescence occurs in mid to late autumn (Connor et al., 1994). Furthermore, warming temperatures are causing winter to become shorter in some geographical regions, with frost occurring later in the season (Loe et al., 2021; Sparks & Menzel, 2002; Williams et al., 2015) and leaf herbivory extending into early autumn (Gallinat et al., 2015; Loe et al., 2021; Zani et al., 2020), such as with caterpillars, leaf miners, and gall wasps (Ekholm et al., 2019, 2022). For example, Lemoine et al. (2014) found that the feeding rates of 11 herbivore‐plant pairs (e.g., *Atteva aurea* and *Ailanthus altissima*, *Danaus plexippus* and *Asclepias syriaca*, among others) increased with warming temperatures. In simulated warming experiments, cumulative consumption for 10 herbivore‐plant pairs increased by 20% over the course of a summer (Lemoine et al., 2014). In some cases, herbivores respond quicker to warmer temperatures compared to their host plants (Burkepile & Parker, 2017; de Sassi & Tylianakis, 2012; DeLucia et al., 2012; Lu et al., 2013). For example, while alligator weed (*Alternanthera philoxeroides*) does not grow more from elevated temperatures, leaf beetles (*Agasicles hygrophila*) feeding on that particular plant overwinter in terrestrial habitats and expand their ranges (Lu et al., 2013). Late‐season defoliation can damage a tree as it affects the accumulation of energy reserves for the leafless period, resulting in less tolerance for colder temperatures and insufficient energy for dormant‐season respiration or new growth in the next growing season (Gregory et al., 1986; Gregory & Wargo, 1986). A pattern has already been observed in over a decade (Ekholm et al., 2019; Liu et al., 2011; Sparks & Menzel, 2002; Vitasse et al., 2009), illustrating the need to expand plant herbivory studies into autumn as well (Gallinat et al., 2015; Williams et al., 2015). One approach to better understand how plants adapt to such changing environmental patterns is to compare past literature on plants chemical defense in response to early autumn seasonality versus herbivory.

### 1.2 Our study

Our meta‐analysis aimed to explore what an overall response in PSM production looks like in a plant, i.e., if a shared effect could be detected across all PSMs expressed in response to herbivory or autumn seasonality in temperate and subtropical regions. Here, the effects of plant type, increase in time since herbivory, precipitation, and temperature were also compared across all PSMs expressed to delve further into how herbivory and autumn seasonality change overall PSM production. Upscaling functional attributes, such as the shared effect of across all PSM production, could be ecologically meaningful for better predicting future environmental dynamics or scenarios, such as how overall PSM production might change with particular temperature increases, herbivory pressures, or in plant types. Our meta‐analysis also measured differences between the largest metabolic subgroups in the dataset to account for functional differences. We expected that overall PSM production would increase from herbivory and seasonality pressure, due stressors such as colder temperatures and herbivory attacks driving induced PSM responses. However, we also expected variation between metabolic subgroups due to them having different functions in the plants.

We focused on answering the following questions:

1. What is the shared effect of herbivory versus early autumn seasonality on all PSM production in a plant?
2. How do woody and herbaceous plant types interact with herbivory and early autumn seasonality to determine PSM production?
3. How does PSM production change with time since herbivory increases?
4. How do changes in precipitation and temperature from summer to early autumn seasonality interact with PSM subgroups to determine production?

## 2 METHODS

### 2.1 Literature search and screening

We chose our response variable as overall PSM production across 22 different PSM subgroups. Here, we also recorded other explanatory variables that can concurrently influence metabolite production in our dataset, including plant type (e.g., herbaceous plants exhibit different PSM production from woody plants), time following herbivory (e.g., PSM production changes from minutes to hours to days), and temperature/precipitation (e.g., plants change PSM production to survive temperature and/or precipitation changes from summer to autumn). We measured these response variables with explanatory variables being autumn seasonality (summer to autumn) and herbivory (absence to presence). Then, excluding subgroups found in fewer than six studies and/or six observations, we were able to also analyze five phenolic PSM subgroups: hydroxybenzoic acids, flavan‐3‐ols, flavonols, hydrolysable tannins, and condensed tannins (Table 1). Other factors, such as UV radiation, light exposure, elevation, and herbivory rate, also play an important role but were not included in this analysis owing to insufficient information in the published studies.

To find published papers meeting the inclusion criteria (described below), the first author searched the Web of Science core collection between April–December 2020 and April–August 2021 (search bouts were separated due to delays resulting from COVID‐19 protocols). The topic search string for season was TS = (secondary metabolite* AND seasonal variation AND leaf) OR TS = (secondary metabolite* AND temporal AND variation* AND leaf) OR TS = (secondary compound* AND seasonal variation AND leaf) OR TS = (secondary compound* AND temporal AND variation* AND leaf). The topic search string for herbivory was TS = (secondary metabolite *AND herbivor* AND leaf) OR TS = (secondary compound* AND herbivor* AND leaf).

These searches retrieved 633 papers (152 for autumn seasonality 481 for herbivory) before further screening (Figure S1). All retrieved papers were screened for eligibility by the first author in two stages, based on the inclusion criteria described below. The titles and abstracts of studies were uploaded into Rayyan software (Ouzzani et al., 2016), and clearly ineligible papers were removed through abstract screening. Following abstract screening, 438 potentially eligible papers underwent full‐text screening. In addition, from April to August 2021, the first author performed a backward and forward search by screening all cited papers in the first 10 paper results from our original search string that met the inclusion criteria, as well as the papers that had cited these same first 10 paper results. This additional search yielded 851 additional papers, with 262 potentially eligible papers going on to full‐text screening. After completing all screenings, 48 papers from the Web of Science search and 11 papers from the backwards and forwards search were retained, with a total of 594 effect sizes from 58 papers (26 for early autumn seasonality, 31 for herbivory, 1 for both study types) used for the meta‐analysis (Figure S1).

### 2.2 Inclusion criteria

#### 2.2.1 Seasonality studies

To be included in the meta‐analysis, a study had to assess the effects of either herbivory or early autumn seasonality on any PSM concentrations measured in leaf dry weight. Seasonality studies compared data recorded in the summer (June–July for Northern hemisphere, December–January for Southern hemisphere) to data recorded in early autumn (September–October for Northern hemisphere, April–May for Southern hemisphere). We excluded studies done in tropical climates (i.e., rainy season/dry season), as the specified months were not comparable to temperate and subtropical climates with autumn seasonality.

#### 2.2.2 Herbivory studies

Herbivory studies compared data recorded from plants in the absence versus presence of leaf‐feeding herbivores (insects and non‐insects). We would have liked to compare herbivory studies only conducted in early autumn for temperate and subtropical regions; however, we were unable to find enough literature for such an analysis. Therefore, we did not place a seasonal or geographical limit on our herbivory studies, including all regions (including tropical), from leaf samples that were taken in nature, in greenhouses, and laboratory. We tested the impact of either study type conducted in greenhouse, laboratory, and nature and did not find an effect (Table S1). By contrast, we excluded papers in which experiments were (1) performed on cell plates, (2) using plants under water, (3) with other plant tissues (e.g., flower, root, bark, and other plant parts), (4) with leaves not collected directly from the plant (e.g., litter), (5) herbivory simulated by humans (e.g., clipping), (6) measuring volatile organic compound emissions (chemicals released into air by plants), and (7) not measuring PSM concentrations in dry weight.

### 2.3 Data collection and extraction

All data were extracted by one author (LS). Extracted descriptive statistics for herbivory and seasonality studies were means, standard deviations, and sample sizes for metabolite content concentration as mg/g of dry weight or in ng/g or μg/g, or “% w/w”; for example, 100 mg of condensed tannin per 100 g of leaf dry weight. PSMs measured in peak area/mg and other similar values were not considered for this study. For papers that provided standard errors only, we converted them to standard deviations using standard equations in Microsoft Excel. For papers that did not report the necessary numerical values, we extracted datapoints from figures using WebPlotDigitizer‐4.2 software (Rohatgi, 2020), standardizing the plot size (through screenshots) and point extraction throughout the analysis.

Additional explanatory variables were recorded for each study. First, although 22 metabolic subgroups were recorded to characterize overall PSM production (Table S2), we further compared differences between five metabolites that had at least 6 effect sizes available in our data: hydroxybenzoic acids, flavan‐3‐ols, flavonols, hydrolysable tannins, and condensed tannins. These five phenolic PSM subgroups have different chemical structures and properties (Table 1). Second, we compared plant types as “woody” or “herbaceous” in our data (e.g., *Quercus robur* was designated as “woody”, and *Geranium sylvaticum* was designated as “herbaceous”).

In addition, we wanted to analyze factors of herbivory and seasonality studies: for herbivory literature, we compared herbivore taxonomic class, insect taxonomic order, and time after herbivory (+24, +48, +72, +120, and +200 h since the beginning of an experiment, with the control – no herbivory – represented by time 0). For autumn seasonality, we compared temperature and precipitation differences between summer and early autumn using study locations to extract information on weather conditions through the *rnoaa* package (version 1.4.0; National Oceanic and Atmospheric Administration) in R (version 4.0.2). These values were from weather stations nearest the reported latitudes and longitudes from each dataset and were summarized from the average of temperature and total precipitation up to 16 days before and including the day of plant collection for a given study.

### 2.4 Effect sizes

We calculated effect sizes (Hedges' *g*) for each comparison (summer vs. early autumn, or herbivore absence vs. presence) with the “escalc” function in the *metafor* package (version 3.8‐1; Viechtbauer, 2010). For herbivory effect sizes, we specified “without herbivory” metabolite concentrations as the numerator and “with herbivory” metabolite concentrations as the denominator, so that positive values indicate that PSM concentrations increased with herbivory, whereas negative values indicate that PSM concentrations decreased with herbivory. For early autumn seasonality effect sizes, we specified June–July and December–January metabolite concentrations as the numerator and September–October or April–May metabolite concentrations as the denominator, so that positive values indicate that PSM concentrations increased from summer to early autumn, whereas negative values indicate that PSM concentrations decreased from summer to early autumn. We applied an exclusion rule to remaining outlier effect sizes higher than typical (effect sizes higher than 5 or less than −5), and their influence on the results was assessed by removing them from the data and re‐running the analyses. We found results to only be influenced by outliers higher than 15 or −15 in new analyses re‐run. Any outlier Hedges' *g* effect sizes higher than typical (effect sizes higher than 15 or less than −15) were also identified as potential data errors and authors were contacted to verify data.

### 2.5 Statistical analyses

We fitted meta‐analytic random effect models using the “rma.mv” function using the restricted maximum likelihood method in the *metafor* package. Statistical significance was denoted by 95% confidence intervals not crossing zero. Random effects were included for observation (i.e., effect size calculated), study, species, and phylogeny to account for non‐independence from multiple observations originating from the same studies and species (Table S3). Species were given using the taxonomic denomination given in papers (most often species but sometimes also intraspecific variants), and plant phylogeny was matched from Open Tree Taxonomy using the *rotl* package (version 3.0.12; Michonneau et al., 2016). The meta‐analytic (intercept‐only) model showed a high heterogeneity of 92.0%, like other ecology meta‐analyses (Senior et al., 2016).

We next used meta‐regression models to explore explanatory variables that could account for some of the unexplained variation between studies and species. For the whole dataset (both study types – herbivory and early autumn seasonality), we first examined the effect of study type: that is, is overall PSM production affected more by the presence of herbivory or early autumn seasonality (Table 2, Model 1). Second, we added an interaction between plant types (woody and herbaceous) and study type from the first model measured (Table 2, Model 2). Third, we added the interaction between study type and phenolic PSM subgroup (Table 2, Model 3). Results from meta‐regressions of explanatory variables were visualized with the *orchaRd* package (version 2.0; Nakagawa et al., 2020).

**TABLE 2:** Models used throughout analysis of herbivory and early autumn seasonality effects on change in plant secondary metabolism production (mg/g of dry leaf), where the response variable is Hedges' *g*.

Number	Data	Description	Explanatory variables
Model 1	Herbivory + autumn seasonality	All PSM production for each study type (autumn seasonality, herbivory)	Study type
Model 2	Herbivory + autumn seasonality	All PSM production between three plant types for each study type (autumn seasonality, herbivory)	Study type, plant type, and their interaction
Model 3	Herbivory + autumn seasonality	PSM production between five phenolic PSM subgroups for each study type (autumn seasonality, herbivory)	Study type, phenolic PSM subgroup, and their interaction
Model 4	Herbivory studies only	All PSM production for increased time since herbivory	Time since herbivory
Model 5	Autumn seasonality studies only	All PSM production for temperature data	Temperature, phenolic PSM subgroup, and their interaction
Model 6	Autumn seasonality studies only	All PSM production for precipitation data	Precipitation, phenolic PSM subgroup, and their interaction

*Note*: All models used the following random effects: Observation ID + study ID + plant species + plant phylogeny. Statistical results are shown in Table S3.

Finally, we ran meta‐regressions on studies of herbivory and season separately (not excluding studies common to both types), thus allowing assessment of explanatory variables that were not applicable to both types of effects. For the herbivory dataset, we ran a univariate meta‐regression examining the effects of hours after herbivory occurred (Table 2, Model 4). For the autumn seasonality dataset, we ran separate meta‐regressions for the effects of temperature and precipitation between summer to early autumn seasonality, including an interaction with phenolic PSM subgroups (Table 2, Models 5 and 6).

### 2.6 Sensitivity analyses

We performed sensitivity analyses to assess how robust our main results were, as recommended in the PRISMA 2020 reporting guideline and checklist (Page, McKenzie, et al., 2021; Page, Moher, et al., 2021). For this, we created funnel plots to look for asymmetry in the data using effect sizes against their inverse standard errors (Figure S2) and performed a time‐lag regression test (Nakagawa et al., 2022) to determine whether studies with larger effects tend to be published earlier by including publication year as an explanatory variable in meta‐regression models (Figure S3, Table S4).

## 3 RESULTS

### 3.1 Description of dataset and heterogeneity

The final dataset included 594 effect sizes from 58 studies, representing 90 different plant species and intraspecific variants. Overall, 55% (33 studies) of the dataset represented herbivory and 45% (27 studies) represented summer to early autumn seasonality (Figure S4, Table S5). PSM categories included 76% (46 studies) representing phenolic PSM subgroups, followed by 13% (11 studies) terpenoids, 8% (four studies) nitrogen‐and‐sulfur containing compounds, and 3% (six studies) nitrogen‐containing compounds (Table S2). Because flavan‐3‐ols, flavonols, hydroxybenzoic acids, condensed tannins, and hydrolysable tannins made up most of our phenolic PSM subgroups (Table S6), we further analyzed the distribution of sample sizes within these five largest phenolic PSM subgroups. Within herbivory, 13% (eight studies) of represented non‐insect herbivory, while 87% (27 studies) represented insect herbivory (Figure S5, Table S7), the insect orders making up most of the dataset being 66% *Lepidoptera* (19 studies), 19% *Hemiptera* (four studies), and 8% *Hymenoptera* (one study) (Figure S6, Table S8). The meta‐analytic mean for the whole dataset was 0.147 (CI: −0.161 to 0.456), which corresponds to the average standardized change in all PSM production from all studies in the analysis.

### 3.2 Effects of herbivory versus early autumn seasonality on overall PSM production

When considering the effects of herbivory and early autumn seasonality on all PSM subgroups, we found no change in overall PSM production in response to herbivory (estimate: 0.345, CI: −0.026 to 0.715, *p* = .069, Figure S4, Table S5) or autumn seasonality (estimate: −0.108, CI: −0.513 to 0.296, *p* = .600; difference between herbivory and early autumn seasonality: −0.453, CI: −0.916 to 0.011, Figure S4, Table S5). We further tested the same effects on phenolic PSM subgroups only, as this represented most of our dataset, where we found a marginally significant increase in response to herbivory (estimate: 0.362, CI: −0.010 to 0.733, *p* = .057, Table S6) and no change in response to autumn seasonality (estimate: −0.048, CI: −0.421 to 0.324, *p* = .799, Table S6).

### 3.3 Effects of herbivory versus early autumn seasonality on woody and herbaceous plant PSM production

**FIGURE 1:**
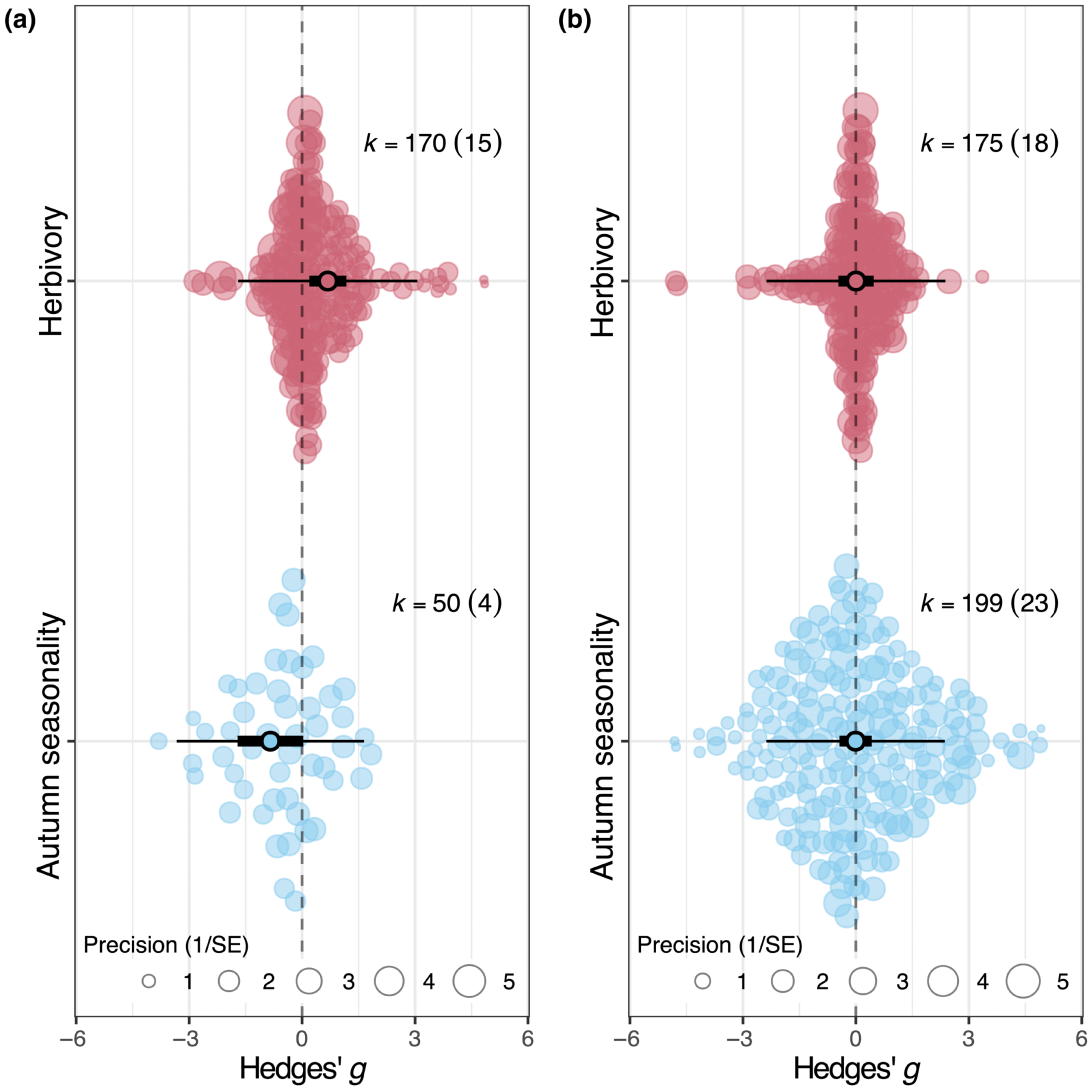
Meta‐regression results for plant secondary metabolism differences between plant type (a – herbaceous, b – woody) and study type (autumn seasonality vs. herbivory). Narrow bars denote prediction intervals, while thick bars denote confidence intervals (95%). *k* is the number of effect sizes included in the analysis. Circle size indicates weight in analysis (inverse of standard error). Whiskers denote 95% confidence intervals; estimates are statistically significant if the confidence intervals do not cross the dashed vertical line. Statistical results are shown in Table S9.

When considering the effects of herbivory on overall PSM production between woody and herbaceous plants, we found an increase in PSM production for herbaceous plants in response to herbivory (estimate: 0.683, CI: 0.193–1.174, *p* = .006, Figure 1, Table S9), while, in contrast, no change in PSM production for woody plants in response to herbivory. Autumn seasonality did not change PSM production between woody and herbaceous plants (estimates ranging from −1.716 in herbaceous plants to 0.419 in woody plants; Figure 1, Table S9).

### 3.4 Effects of increase in time since herbivory and herbivore taxonomic class

**FIGURE 2:**
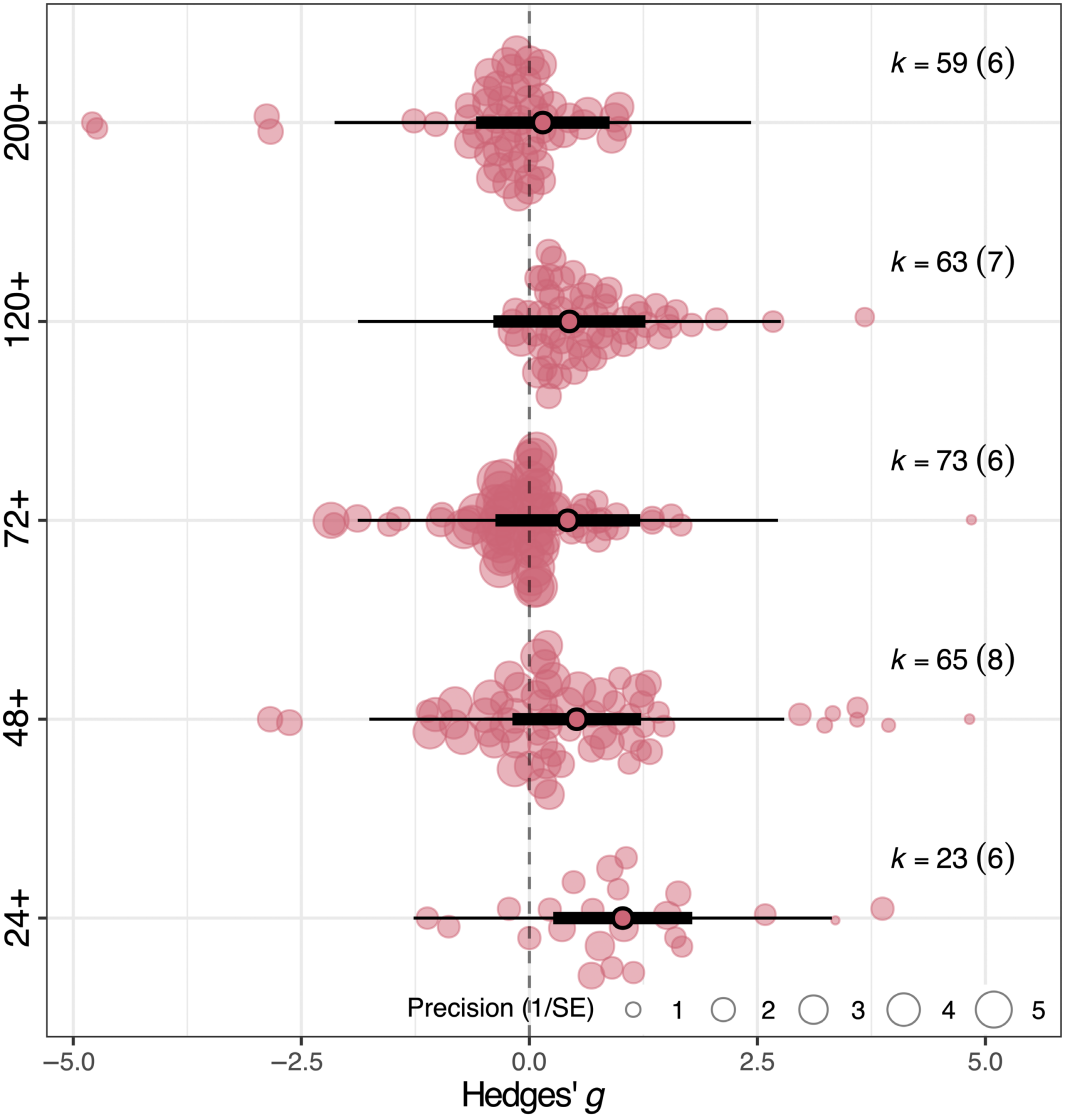
Meta‐regression results for plant secondary metabolism differences between hours after herbivory. Narrow bars denote prediction intervals, while thick bars denote confidence intervals (95%). *k* is the number of effect sizes included in the analysis. Circle size indicates weight in analysis (inverse of standard error). Whiskers denote 95% confidence intervals; estimates are statistically significant if the confidence intervals do not cross the dashed vertical line. Statistical results are shown in Table S10.

When examining the effects of increase in time since herbivory, overall PSM production increased in samples collected 24+ h since herbivory occurred (estimate: 1.024, CI: 0.261–1.786, *p* = .009, Figure 2, Table S10). Overall PSM production did not increase further in samples collected between 48+ and 120+ h after herbivory (estimates ranging from 0.520 to 0.439, Figure 2, Table S10), or in samples collected at 200+ h after herbivory occurred (estimate: 0.148, CI: −0.584 to 0.880, *p* = .693, Figure 2, Table S10) but samples collected at 200+ h after herbivory occurred contrasted negatively with samples collected 24+ h (estimate difference between 24+ and 200+: −0.876, CI: −1.436 to −0.316, *p* = .002, Table S10). We also compared the effects of herbivore taxonomic class and insect taxonomic order to account for feeding guild differences but found overall PSM production did not change in response to the type of herbivory (Figures S5 and S6, Tables S7 and S8).

### 3.5 Effects of herbivory versus early autumn seasonality on individual PSMs within phenolic PSM subgroups

**FIGURE 3:**
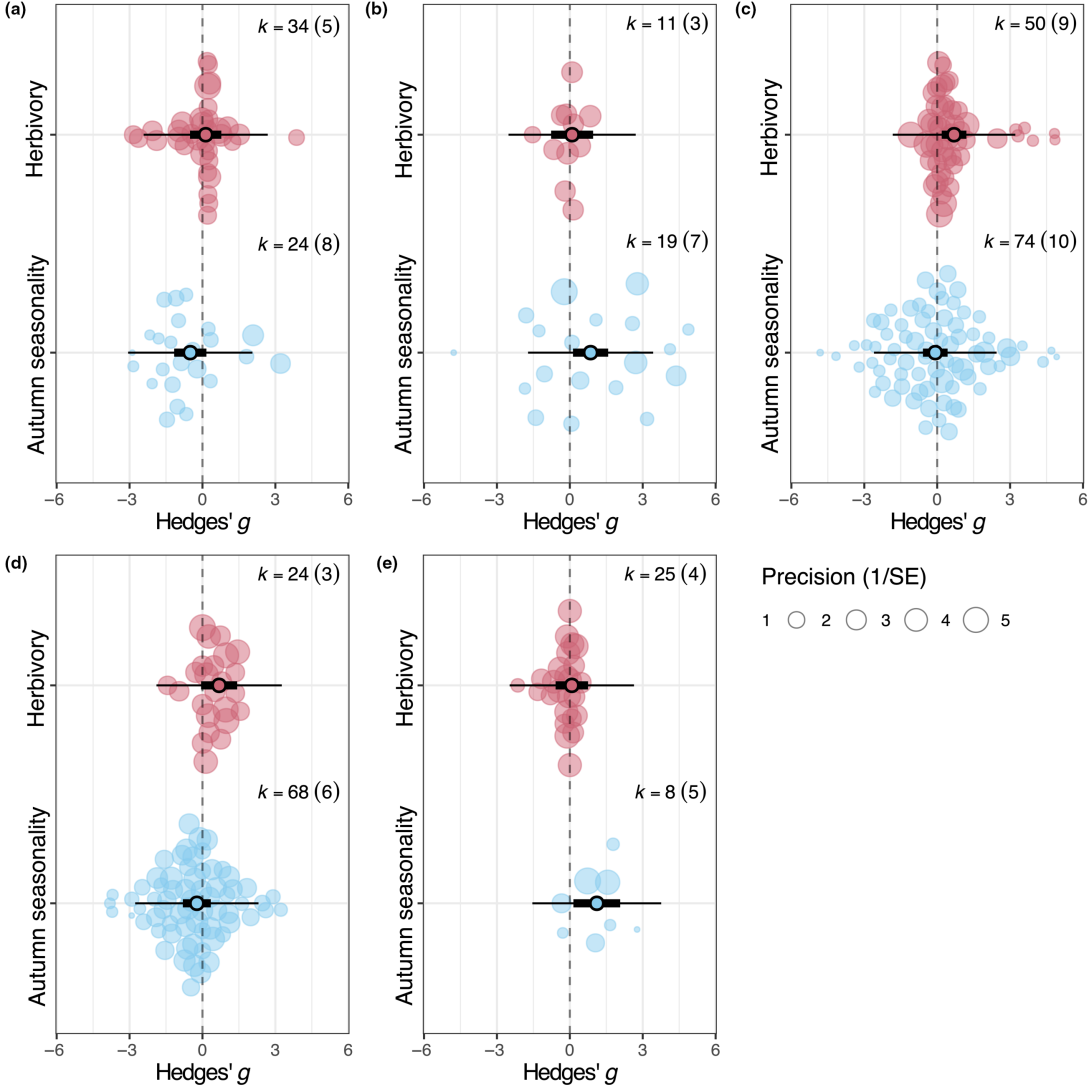
Meta‐regression results for individual phenolic plant secondary metabolism subgroup differences between herbivory and early autumn seasonality measured in papers. Letters correspond to the following: (a) hydroxybenzoic acids, (b) flavan‐3‐ols, (c) flavonols, (d) hydrolysable tannins, (e) condensed tannins. Narrow bars denote prediction intervals, while thick bars denote confidence intervals (95%). *k* is the number of effect sizes included in the analysis. Circle size indicates weight in analysis (inverse of standard error). Whiskers denote 95% confidence intervals; estimates are statistically significant if the confidence intervals do not cross the dashed vertical line. Statistical results are shown in Table S11.

When examining the effects of herbivory within phenolic PSM subgroups, an increase in flavonol was observed (estimate: 0.693, CI: 0.185–1.202, *p* = .008, Figure 3c, Table S11). By contrast, the remaining phenolic PSM subgroups (hydroxybenzoic acids, flavan‐3‐ols, hydrolysable tannins, hydrolysable, and condensed tannins) did not change in response to herbivory (estimates ranging from 0.083 in condensed tannins to 0.685 in hydrolysable tannins; Figure 3, Table S11). When examining the effect of season within phenolic PSM subgroups, condensed tannin increased from summer to early autumn (estimate: 1.110, CI: 0.148–2.071, *p* = .024, Figure 3e, Table S11), as well as flavan‐3‐ol (estimate: 0.855, CI: 0.131–1.578, *p* = .021, Figure 3b, Table S11). The remaining phenolic PSM subgroups (hydroxybenzoic acids, flavonols, and hydrolysable tannins) did not change from summer to early autumn (estimates ranging from −0.502 in hydroxybenzoic acids to −0.078 in in flavonols, Figure 3, Table S11).

### 3.6 Effects of precipitation and temperature

**FIGURE 4:**
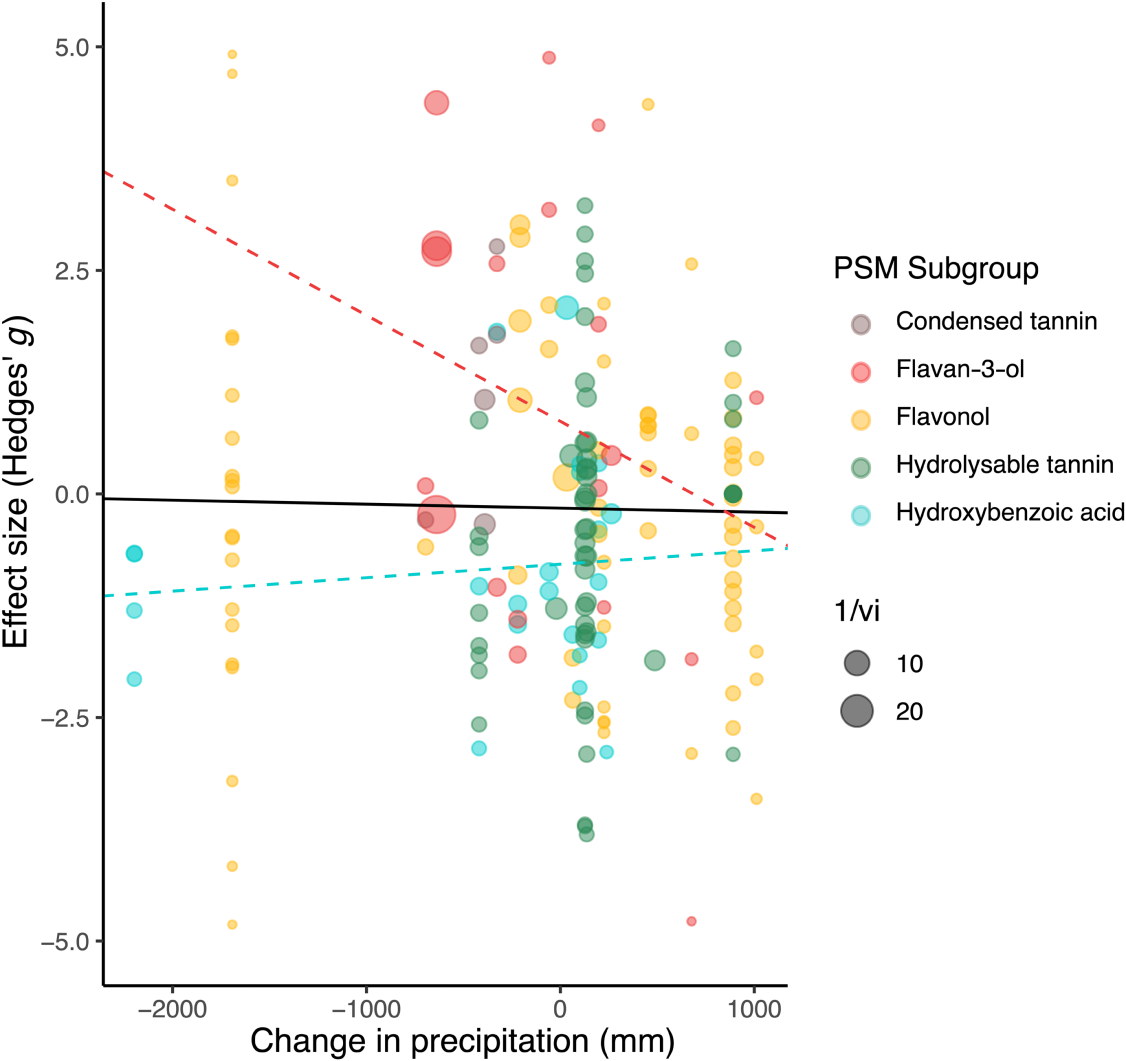
Meta‐analytic results for plant secondary metabolism differences with changes in precipitation. Circles indicate effect sizes of five largest metabolic subgroups in dataset and circle size indicates weight in analysis (inverse of standard error). Dashed lines show marginally significant intercept and slope estimates for flavan‐3‐ol and hydroxybenzoic acid meta‐regression models. Black line indicates the slope for all 22 metabolic subgroups found in dataset (Table S2). Statistical results are shown in Table S12.

**FIGURE 5:**
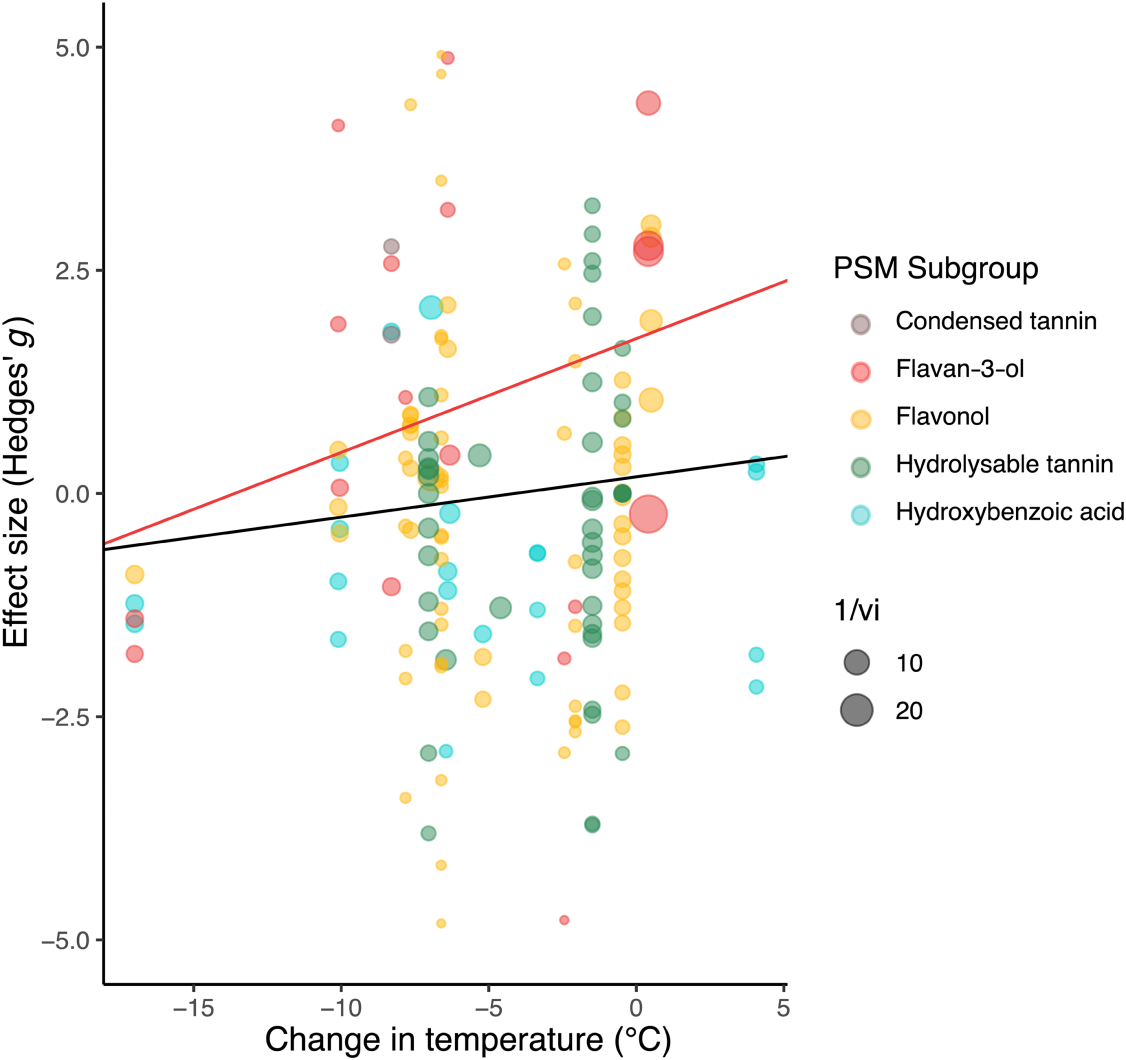
Meta‐analytic results for plant secondary metabolism differences with changes in temperature. Circles indicate effect sizes of five largest metabolic subgroups in dataset and circle size indicates weight in analysis (inverse of standard error). Red line shows significant intercept and slope estimates for flavan‐3‐ol meta‐regression models. Black line indicates the slope for all 22 metabolic subgroups found in dataset (Table S2), while red slope indicates flavan‐3‐ol production, which decreased as temperature lowered from summer to fall (*p* = .016). Statistical results are shown in Table S12.

When examining the effects of precipitation within the five phenolic PSM subgroups collected between summer to early autumn, flavan‐3‐ol production exhibited a marginally significant increase following precipitation (estimate: 0.812, CI: −0.016 to 1.640, *p* = .054, Figure 4, Table S12), while hydroxybenzoic acid production showed a marginally significant decrease following precipitation (estimate: −0.786, CI: −1.587 to 0.015, *p* = .055, Figure 4, Table S12). The remaining phenolic PSM subgroups (flavonols, hydrolysable tannins, and condensed tannins) did not change in response to precipitation (estimates ranging from −0.594 in hydrolysable tannins to 3.541 in condensed tannins, Figure 4, Table S12). When examining the effects of temperature, an increase in flavan‐3‐ol production was also observed with higher temperatures (estimate: 1.736, CI: 0.322–3.149, *p* = .016, Figure 5, Table S12), while the remaining phenolic PSM subgroups (flavonols, hydrolysable tannins, and condensed tannins) did not change in response to temperature (estimates ranging from −0.634 in hydrozybenzoic acids to 2.309 in condensed tannins, Figure 5, Table S12).

### 3.7 Publication bias and sensitivity analyses

Visual inspection of funnel plots indicated some asymmetrical distribution of effect sizes around the meta‐analytic mean (Figure S2A,B). When averaging the effect sizes within studies, asymmetry was observed, with a few moderately sized studies falling outside of the funnel shape and having statistically significant effect sizes (gray areas, Figure S2D). However, the results of the time‐lag regression test on the meta‐analytic residuals showed no presence of publication bias in the dataset (Table S4, Figure S3).

## 4 DISCUSSION

Using meta‐analysis, we were able to compare effects of herbivory (absence to presence) and autumn seasonality (summer to early autumn) on overall PSM production across multiple PSM subgroups. We next discuss herbivory and seasonality impacting PSM production differently, including (1) how herbivory increases overall PSM production in herbaceous plants, (2) how overall PSM production increases with time following herbivory yet persists, and (3) how important variation also exists among phenolic PSM subgroups that could be season‐dependent, or (4) due to plants producing different PSMs when resources for growth and reproduction become less available. Finally, we discuss how (5) more studies are required to help us elucidate how autumn seasonality affects plant–herbivore interactions. Below we describe the detected patterns in more detail and considered possible explanations with suggestions for future research.

### 4.1 Herbivory increases overall PSM production in herbaceous plants

By comparing effects of herbivory and early autumn seasonality on overall PSM production, we gain a broader understanding of how plants cope with two different events. We predicted that overall PSM production would compositionally change with the presence of herbivory based on previous traditional assertions that plants typically ramp up chemical defenses in response to herbivory (Bennett & Wallsgrove, 1994; Pichersky & Gershenzon, 2002; Swain, 1977). Our results indeed seem to suggest that rather herbivory but not seasonality has a positive effect on an herbaceous plant's PSM production. Still, seasonality can indirectly cause changes to PSM production, such as how some herbaceous plants, such as alligator weed, are heavily impacted by an increased herbivory rate due to increased temperatures, such as found in leaf beetles (Lu et al., 2013).

Perhaps a reason for why woody plants do not increase their PSM production in response to herbivory is they store more nutrients in bark and roots and rely on compensatory growth as an alternative strategy to respond to herbivory (Boeckler et al., 2013; Haukioja & Koricheva, 2000). In addition, woody plants can constitutively produce PSMs, while herbaceous plants only have a limited reserve of nutrients and fewer resources for storage and rely on induced responses (Boeckler et al., 2013; Haukioja & Koricheva, 2000). Also, induced responses can be systemic, altering PSM production throughout the whole plant (Kachroo & Robin, 2013), or they can be localized, for example altering PSM production only on the branch where herbivore attack has occurred, potentially reducing the metabolic cost to the larger plants (Volf et al., 2021). Systemic‐induced responses have been found to take longer to spread in larger woody species than in annual herbaceous plants, i.e., a signal is locally generated at the damage site and then spreads, meaning smaller herbaceous plants can respond faster to herbivory with less lag (Gutbrodt et al., 2011), which perhaps could be reflected here in our results.

### 4.2 Overall PSM production increases initially with time following herbivory, but persists

Our results concur with previous studies demonstrating that induced chemical responses to herbivory are highest initially following herbivory (Agrell et al., 2003; Anderson et al., 2001; Gómez et al., 2010; Schultz, 1989; Underwood, 1998). The rapid induction of chemical defense after herbivory can occur within just a few hours after damage (Alborn et al., 1996; Karban & Baldwin, 2007) and confers a direct benefit to the plant, since herbivores are expelled from the plant, limiting further damage (Mason et al., 2017; Ruuhola et al., 2008). In addition, some plant‐induced responses peak with a time lag of 3–4 days (Anderson et al., 2001; Gutbrodt et al., 2011; Underwood, 1998). The time lag benefits the plant in situations where herbivore pressure gradually increases after the first attack, resulting in the induction of increased PSM production when the greatest number of herbivores are present (Backmann et al., 2019).

However, our results also indicate that overall plant responses can be long‐lived, which concurs with Boeckler et al. (2013) and Gómez et al. (2010) finding PSM responses to herbivory detectable after weeks, although at lower concentrations. It is possible that some PSM responses are constitutively expressed regardless of herbivory damage, and it would be valuable to test in a future study if PSM constitutive responses are higher in seasons, such as autumn and winter, and whether increased herbivory in autumn might affect such mechanisms (i.e., forcing the plant to rely more on induced responses in a season). Martin and Müller (2007) also point out that the duration of induced responses to herbivory depends on metabolite chemical subgroup (Karban & Baldwin, 2007), plant species (Neuvonen, 1987), season (Karban & Baldwin, 2007), and type of attack to which a plant is subjected (Mathur et al., 2011). Herbivore feeding guilds such as gall formers, leaf‐chewers, sap‐feeders, and pitch borers differ in severity of injury, from causing small reductions in plant growth and reproduction (Price et al., 1987) to damaging essential conductive tissue that the plant is unable to replace (Haack & Slansky, 1987). We compared insect orders to inspect potential feeding guild impacts but did not find any differences in effect size between insect orders on PSM production for our results (Table S9, Figure S6).

### 4.3 Variation exists among phenolic PSM subgroups that could be season‐dependent

Condensed tannins play a critical role in plant–herbivore interactions, as they can bind and precipitate proteins as well as act as prooxidants (Barbehenn & Peter Constabel, 2011; Kraus et al., 2003; Salminen & Lempa, 2002). Yet, our results suggest that early autumn seasonality but not herbivory increased condensed tannin production. This result could be due to a couple of explanations. Recent work has highlighted the importance of other biotic and abiotic factors in the production of condensed tannin (Ashraf et al., 2018; Gourlay & Constabel, 2019; Kraus et al., 2003; Li et al., 2020; Salminen, 2018). Typically, phenolic compounds could help plants combat drought (Yang et al., 2018), such as illustrated in a study of *Quercus rubra* in controlled environments, where plants increased condensed tannin production in response to a combination of warmer and drier conditions (Top et al., 2017). Condensed tannins also have inherent properties that protect plants against oxidative stress (Gourlay & Constabel, 2019). Similar patterns might occur for plants experiencing sudden low‐temperature stress. During stress, growth is often inhibited more than photosynthesis, and the carbon that is fixed is predominantly allocated toward the production of defense chemicals (Ramakrishna & Ravishankar, 2011). Moreover, Volf et al. (2022) found condensed tannins to have an elevational increase with higher humidity and longer periods of cold but decreasing with fungal damage. A similar trend might be observed from our results from summer to early autumn as higher precipitation and colder temperatures become more prevalent. As we did not measure moisture levels or fungal damage in trees, we therefore propose this as a future topic to investigate (Box 2).

BOX 2 **Potential questions for future studies**

1. How does seasonal variation in herbivory affect plant PSM production?
2. Do specialist herbivores influence PSM production differently from generalist herbivores with autumn seasonality?
3. How might the inclusion of more metabolic subgroups aside from phenolics, such as alkaloids and glucosinolates, further estimate the “shared” effect of herbivory and autumn seasonality on PSM production?
4. How do latitude and humidity influence plant PSM production with autumn seasonality versus with herbivory?
5. How do deciduous versus evergreen plants differ in coping with herbivory and autumn seasonality?
6. What is the effect of non‐leaf herbivory (i.e., root herbivory, etc.) on PSM production between summer to autumn season?
7. What is the effect of herbivory by different insect guilds (i.e., leaf chewing, sap‐sucking, boring, etc.) on PSM production between summer to autumn season?

Similar to condensed tannins, flavan‐3‐ol production also increased seasonally from summer to early autumn. However, flavan‐3‐ol production decreased when temperatures lowered from summer to early autumn, corroborating previous research reporting low‐temperature stress causes a decrease in the biosynthesis and storage of some PSMs (Ashraf et al., 2018; Li et al., 2020; Verma & Shukla, 2015). As precursors to condensed tannins in the same biosynthetic pathway (Table 1), a presence of flavan‐3‐ols could be a trace indicator of plants synthesizing condensed tannins during early autumn, or simply that flavan‐3‐ols are advantageous for plants in some capacity during autumn, similarly to condensed tannins.

### 4.4 Plants produce different PSMs when resources for growth and reproduction become less available

In contrast, our results depicted flavonol production to be increased by herbivory but not seasonality. Previous studies found that flavonol production increases following herbivory (Chin et al., 2018; Mierziak et al., 2014), as flavonols act as deterrents against herbivory and have cytotoxic impacts on insects, for instance by inhibiting development and increasing mortality (Mierziak et al., 2014). In addition, unlike condensed tannins, these metabolites require less energy to be produced (Strack, 1997) and can therefore be induced more readily in larger concentrations following herbivory, regardless of the environment and season. Flavonols also protect plants from UV radiation (Ferreyra et al., 2021; Yang et al., 2018) and play other roles in plant primary metabolism while occurring at low concentrations (Pollastri & Tattini, 2011). With UV radiation declining in autumn, a downward trend might be expected for flavonol production in response to seasonality changes. However, since flavonol plays an important role in plant primary metabolism while occurring at low concentrations (Pollastri & Tattini, 2011), it is possible that production does not shift much, if at all, by early autumn seasonality, such as seen in our results. This observation also supports the idea that plants can both constitutively express a PSM, such as flavonol, in small amounts for primary functions, while still having induced responses of that same PSM when experiencing herbivory. In that sense, increased flavonol production following herbivory also benefits the plant's growth and other primary functions once the herbivores are absent (McCall & Fordyce, 2010). Our results fit the growth‐differentiation balance hypothesis (GDB) describing how plants allocate between differentiation and growth‐related processes in various environmental conditions (Stamp, 2004). According to the GDB hypothesis, competition favors allocation tradeoffs to growth, while herbivory favors allocation tradeoffs to secondary metabolism (Herms & Mattson, 1992). Resource allocation trade‐offs associated with plant growth and defense might explain the observed pattern here in that the intensity of selection for defenses that deter herbivory increases as the availability of resources in the environment declines (Bryant et al., 1992). Another consideration is that these trade‐offs occur because sharing the same metabolic pathway could cause competition for precursor metabolites during synthesis of specialized metabolites. Still, like other plant defense hypotheses (e.g., carbon‐nutrient hypothesis, resource availability hypothesis), the GDB hypothesis is not always applicable, such as when the probability of herbivore attack or value of plant tissue varies (Hamilton et al., 2001). Therefore, we believe it is important to test the gaps in the GDB and other resource hypotheses for future meta‐analytic studies on autumn PSM production.

### 4.5 More studies are required to help us elucidate how autumn seasonality affects plant–herbivore interactions

Overall PSM production by plants is affected by different pressures in some capacity, such as herbivory and autumn seasonality, yet we lack documentation on several metabolic subgroups to fully detect any shared effect across all metabolites. While we did not detect a shared signal across all PSMs or phenolic PSM subgroups in response to herbivory or early autumn seasonality when comparing all plant types, we suspect that if more data were available on other PSM subgroups belonging to terpenoids, nitrogen‐containing, and nitrogen‐and‐sulfur‐containing groups, we might detect interesting patterns and make comparisons outside phenolic PSM subgroups (Table S2). In addition, our herbivore dataset was *Lepidoptera* dominated (Figure S6), which is relevant when considering that the hydrolysis of tannins producing gallic acid is relatively non‐toxic for many caterpillar guts (Barbehenn & Peter Constabel, 2011). It might explain why no trend was found for tannins in response to herbivory, and that more data on other types of herbivory, such as mammalian, would be beneficial. Finally, we would have liked to directly compare the effects of herbivory on PSM production in studies conducted during early autumn. However, most herbivory studies are either conducted in spring or summer, near the equator, averaged across 1 year, or conducted under controlled greenhouse conditions, indicating a lack of literature on plant–herbivore interactions during early autumn. We therefore encourage future investigations on interactions between PSM production and herbivory during autumn, specifically focusing on chemical defenses beyond phenolic PSM subgroups, in addition to other potential factors that should be further analyzed (Box 2).

## AUTHOR CONTRIBUTIONS

**L. Skovmand:** Conceptualization (lead); data curation (lead); formal analysis (equal); funding acquisition (supporting); investigation (equal); methodology (equal); project administration (lead); resources (lead); software (lead); supervision (lead); validation (equal); visualization (lead); writing – original draft (lead); writing – review and editing (lead). **Rose E. O'Dea:** Data curation (supporting); formal analysis (equal); investigation (equal); methodology (equal); validation (equal); writing – review and editing (supporting). **Keri A. Greig:** Conceptualization (supporting); writing – original draft (supporting); writing – review and editing (equal). **Katherine R. Amato:** Conceptualization (supporting); investigation (supporting); visualization (supporting); writing – review and editing (supporting). **Andrew P. Hendry:** Conceptualization (supporting); formal analysis (supporting); funding acquisition (lead); investigation (supporting); visualization (supporting); writing – review and editing (supporting).

## CONFLICT OF INTEREST STATEMENT

The authors have no relevant financial or non‐financial interests to disclose.

### OPEN RESEARCH BADGES

This article has earned Open Data and Open Materials badges. Data and materials are available at the full data including search, extracted, and author data and R scripts are available on OSF https://doi.org/10.17605/osf.io/5wt6p; https://archive.org/details/osf‐registrations‐sy8d6‐v1.

## Supporting information


Appendix S1.

## Data Availability

Data, analysis script, and lists of screened studies are available to download from https://osf.io/wrj6v/?view_only=58246da73fde4d7ba4a5dd2d6d293962.
