# Supplementary material for: Effects of leaf herbivory and autumn seasonality on plant secondary metabolites: A meta‐analysis
Source: Ecol Evol. 2024 Feb 13;14(2):e10912. doi: 10.1002/ece3.10912 (PMC10864732; doi:10.1002/ece3.10912)
Supplement: Supplementary file 1 — Appendix S1. [file ECE3-14-e10912-s001.docx]

**Supporting Information for:**

# Effects of leaf herbivory and autumn seasonality on plant secondary metabolites: a meta-analysis

Authors: ^1^Skovmand, L., ^2^O’Dea, R.E., ^3^Greig, K.A. ^4^Amato, K.R., ^1^Hendry, A.P.

^1^Redpath Museum & Department of Biology, McGill University, Montreal, Quebec, Canada

^2^School of Ecosystem and Forest Sciences, University of Melbourne, Melbourne, Australia

^3^Department of Biology, University of Texas, Austin, USA

^4^Department of Anthropology, Northwestern University, Evanston, USA


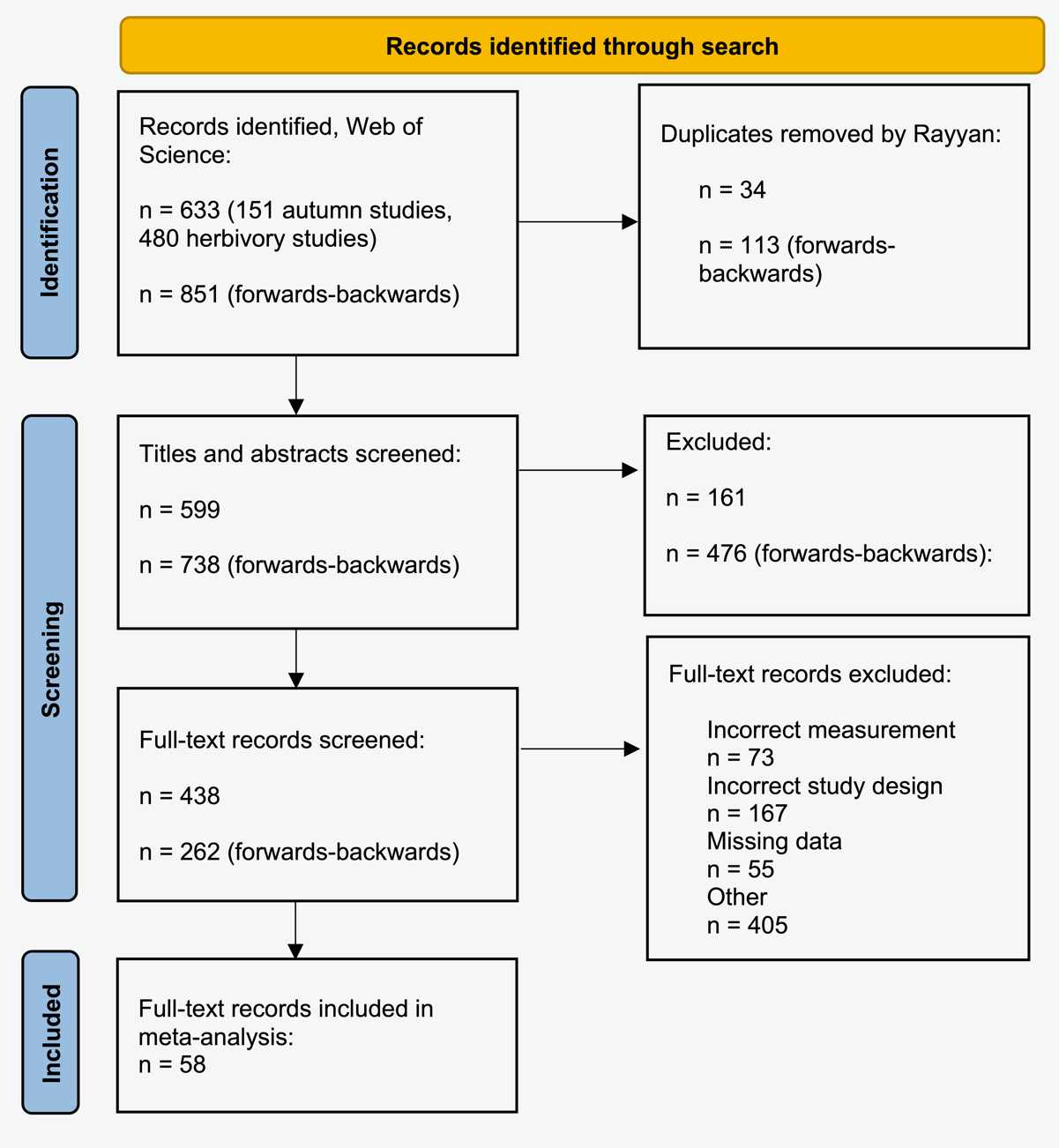


## Figure S1

PRISMA diagram. Modified version of: Page MJ, McKenzie JE, Bossuyt PM, Boutron I, Hoffmann TC, Mulrow CD, et al. The PRISMA 2020 statement: an updated guideline for reporting systematic reviews. BMJ 2021;372:n71. doi: 10.1136/bmj.n71


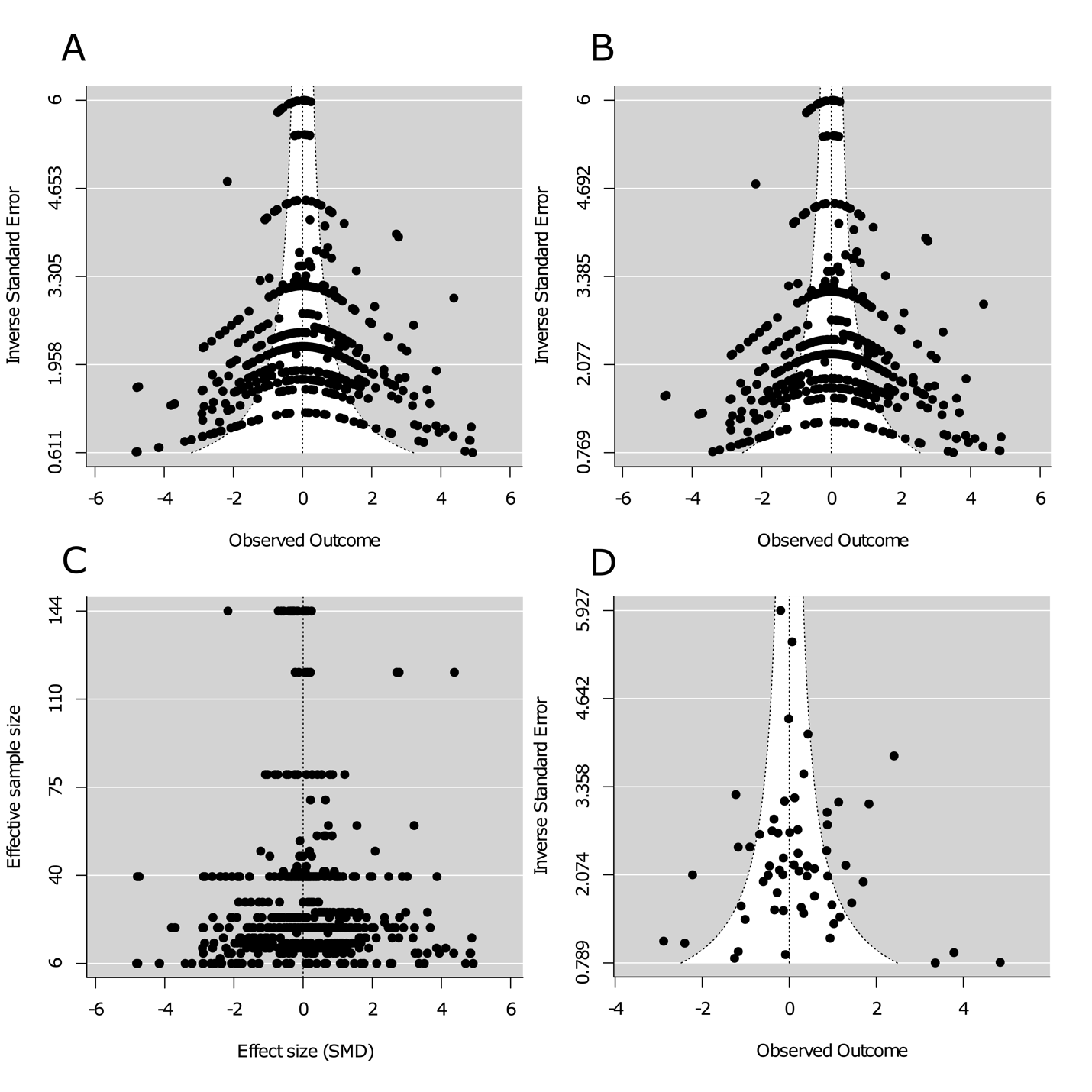


## Figure S2

Funnel plots showing the distribution of effect sizes using A) inverse standard error with observed outcome, B) inverse standard error with observed outcome using a subset of dataset of relatively small variances, C) effective sample sizes with effect size (SMD), and D) inverse standard error with observed outcome for study averages.


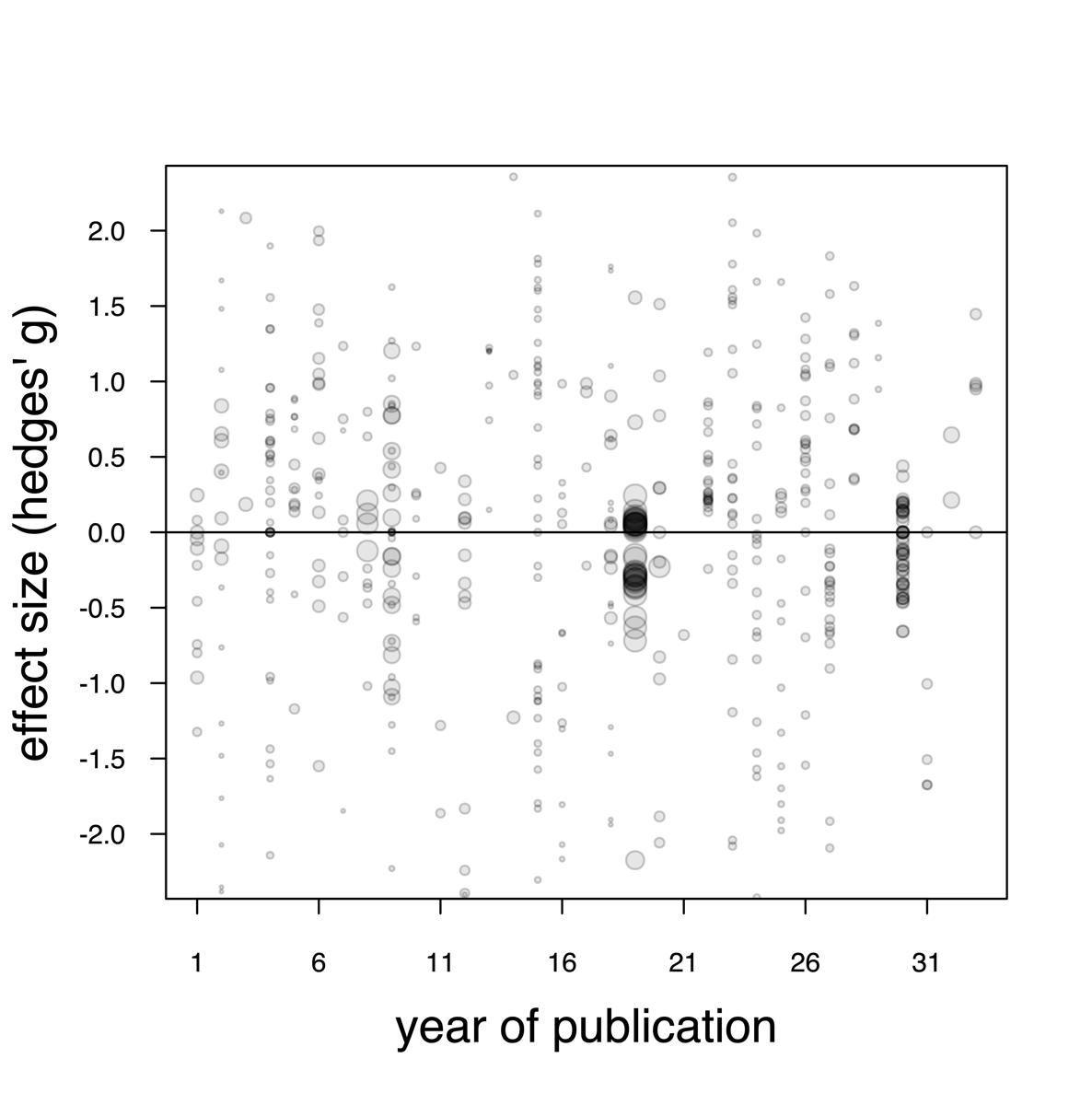


**Figure S3**

Bubble plot showing time-lag regression test on the relationship between effect sizes (Hedges’ g) and year of publication.

**
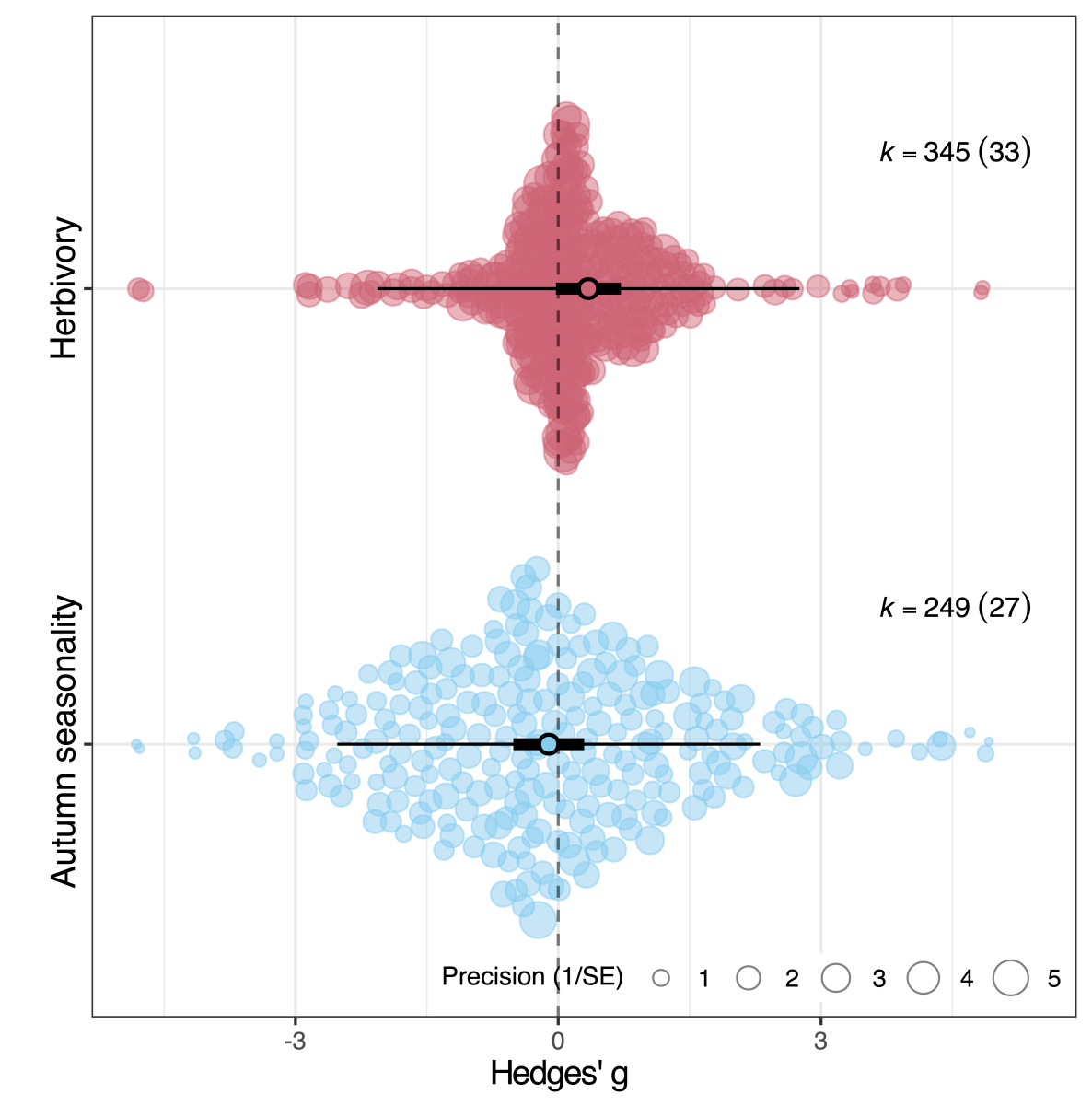
**

**Figure S4**. Meta-regression results for PSM differences (all metabolites included) between herbivory and early autumn seasonality measured in papers. Narrow bars denote prediction intervals, while thick bars denote confidence intervals (95%). *k* is the number of effect sizes included in the analysis. Circle size indicates weight in analysis (inverse of standard error). Whiskers denote 95% confidence intervals; estimates are statistically significant if the confidence intervals do not cross the dashed vertical line. Statistical results are shown in Table S5.


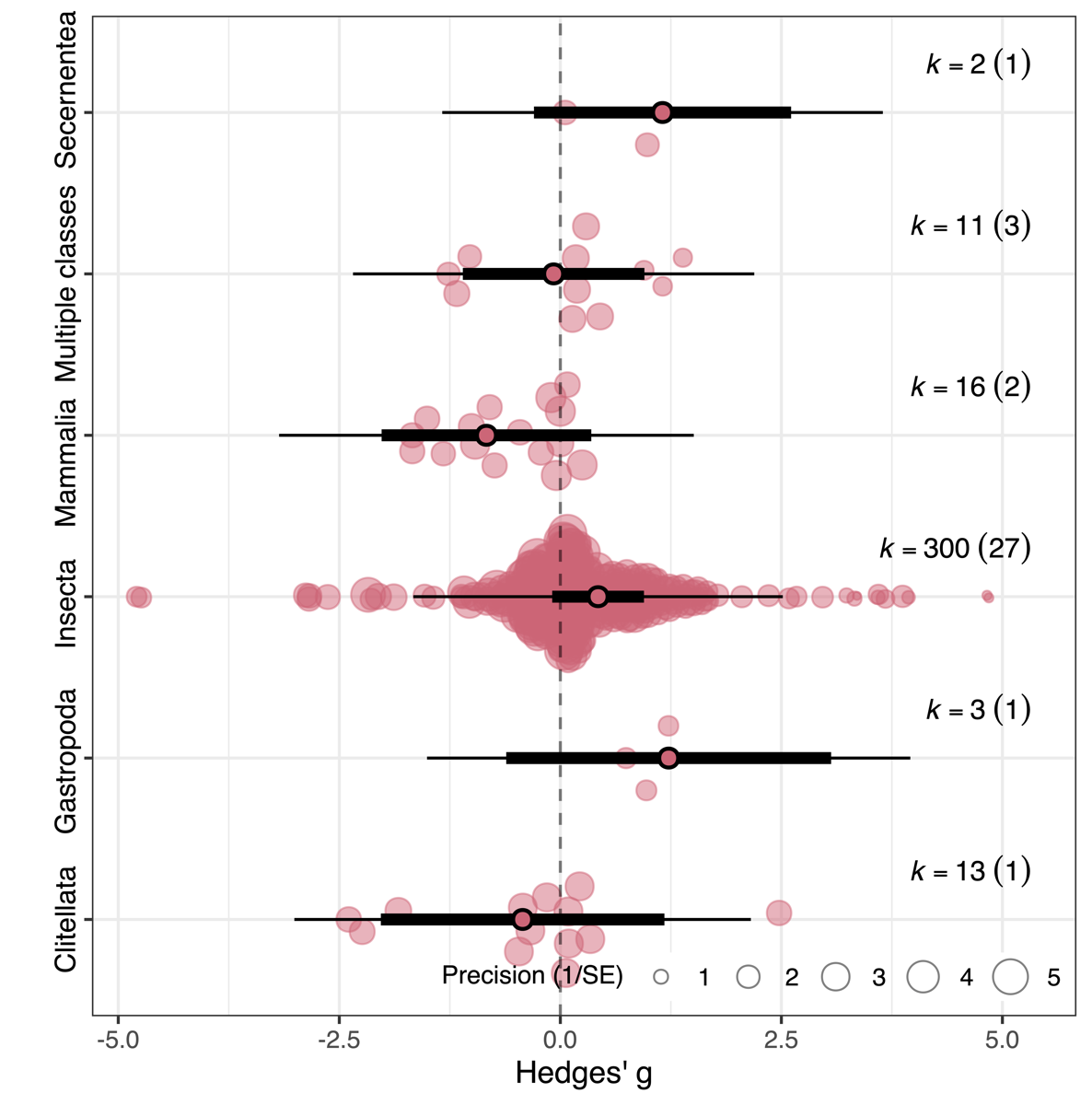


**Figure S5**

Meta-regression results for PSM differences between herbivore classes. Statistical results are found in Table S7 and show that herbivore class in this dataset does not influence PSM production.


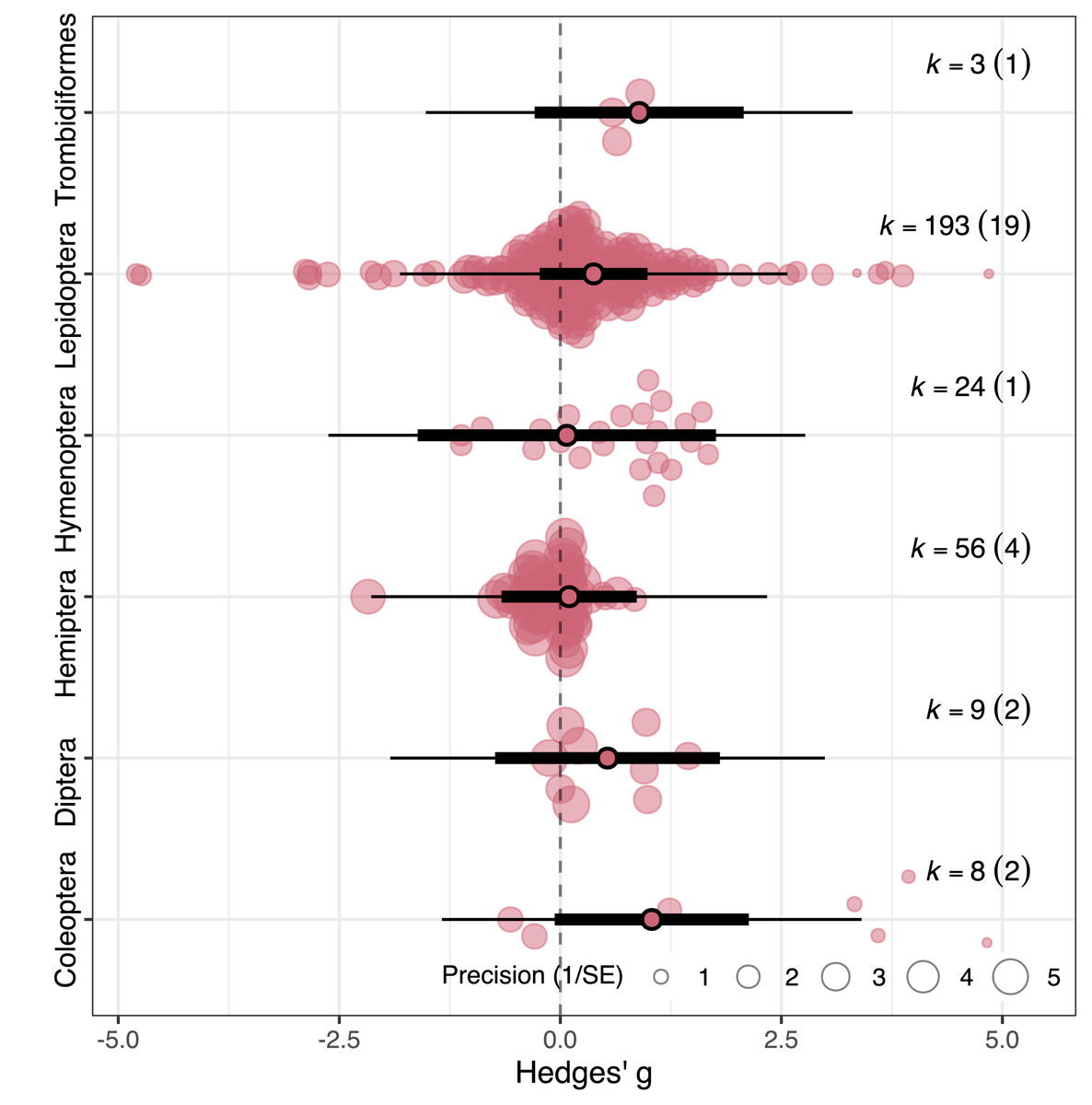


**Figure S6**

Meta-regression results for PSM differences between insect herbivore orders. Statistical results are found in Table S8 and show that herbivore order in this dataset does not influence PSM production.

| **Table S1: Effects of study environment (greenhouse, laboratory, or nature) on typical PSM production.** | | | | | | | |
| --- | --- | --- | --- | --- | --- | --- | --- |
|  |  |  |  |  |  |  |  |
| **Greenhouse** | | | | | | | |
| **PSM subgroup** | **Type of estimate** | **Effect on PSM (hedges' g)** | | | | **Sample sizes** | |
|  |  | Mean est | Lower CI | Upper CI | pval | N studies | k effect sizes |
| all | mean | 0.412 | -0.006 | 0.830 | 0.053 | 19 | 189 |
| all | contrast with laboratory | 0.332 | -0.675 | 1.339 | 0.518 |  |  |
| all | contrast with nature | -0.474 | -0.970 | 0.022 | 0.061 |  |  |
| **Laboratory** | | | | | | | |
| **PSM subgroup** | **Type of estimate** | **Effect on PSM (hedges' g)** | | | | **Sample sizes** | |
|  |  | Mean est | Lower CI | Upper CI | pval | N studies | k effect sizes |
| all | mean | 0.748 | -0.191 | 1.686 | 0.118 | 4 | 40 |
| all | contrast with nature | -0.807 | -1.776 | 0.163 | 0.103 |  |  |
| **Nature** | | | | | | | |
| **PSM subgroup** | **Type of estimate** | **Effect on PSM (hedges' g)** | | | | **Sample sizes** | |
|  |  | Mean est | Lower CI | Upper CI | pval | N studies | k effect sizes |
| all | mean | -0.059 | -0.395 | 0.277 | 0.730 | 37 | 365 |

| **Table S2: PSM subgroups found in dataset.** | | | | |
| --- | --- | --- | --- | --- |
|  |  |  |  |  |
| **PSM subgrop** | **PSM group** | **Study type** | **k effect sizes** | **N studies** |
| alkaloid | nitrogen-containing | autumn seasonality | 5 | 3 |
| alkaloid | nitrogen-containing | herbivory | 9 | 2 |
| amino acid | nitrogen-containing | autumn seasonality | 2 | 1 |
| amino acid | nitrogen-containing | herbivory | 1 | 1 |
| anthocyanin | phenolic | herbivory | 3 | 1 |
| anthraquinone | phenolic | herbivory | 15 | 2 |
| brevifolin carboxylic acid | phenolic | autumn seasonality | 1 | 1 |
| condensed tannin | phenolic | autumn seasonality | 8 | 5 |
| condensed tannin | phenolic | herbivory | 25 | 4 |
| diterpene | terpenoid | autumn seasonality | 7 | 2 |
| diterpene | terpenoid | herbivory | 22 | 3 |
| flavan-3-ol | phenolic | autumn seasonality | 19 | 7 |
| flavan-3-ol | phenolic | herbivory | 11 | 3 |
| flavanone | phenolic | herbivory | 4 | 1 |
| flavone | phenolic | autumn seasonality | 11 | 3 |
| flavone | phenolic | herbivory | 16 | 2 |
| flavonol | phenolic | autumn seasonality | 74 | 10 |
| flavonol | phenolic | herbivory | 50 | 9 |
| flavonol glycoside | phenolic | autumn seasonality | 8 | 2 |
| glucosinolate | nitrogen-and-sulfur-containing | herbivory | 45 | 4 |
| hydrolysable tannin | phenolic | autumn seasonality | 68 | 6 |
| hydrolysable tannin | phenolic | herbivory | 24 | 3 |
| hydroxybenzoic acid | phenolic | autumn seasonality | 24 | 8 |
| hydroxybenzoic acid | phenolic | herbivory | 34 | 5 |
| hydroxycinnamic acid | phenolic | autumn seasonality | 6 | 4 |
| hydroxycinnamic acid | phenolic | herbivory | 1 | 1 |
| lignin | phenolic | autumn seasonality | 3 | 2 |
| lignin | phenolic | herbivory | 7 | 2 |
| monoterpene | terpenoid | autumn seasonality | 5 | 2 |
| monoterpene | terpenoid | herbivory | 16 | 1 |
| salicinoid | phenolic | herbivory | 27 | 2 |
| sesquiterpene | terpenoid | herbivory | 21 | 2 |
| simple phenol | phenolic | autumn seasonality | 8 | 1 |
| simple phenol | phenolic | herbivory | 6 | 1 |
| triterpene | terpenoid | herbivory | 8 | 3 |

| **Table S3: Variance components in intercept-only models.** | | | | | | | |
| --- | --- | --- | --- | --- | --- | --- | --- |
|  |  |  |  |  |  |  |  |
| **Study ID** | | | | | | | |
| model |  | Heterogeneity | | | | AIC | R^2^ (conditional) |
|  |  | mode | mean | sqrt | nlvls |  |  |
| meta-analytic |  |  |  |  |  | 1953 | 0.590 |
|  |  | *I*^2^_study_ID_ | 0.593 | 0.770 | 60 |  |  |
|  |  | *I*^2^_obs_ID_ | 0.868 | 0.932 | 594 |  |  |
| **Species** | | | | | | | |
| model |  | Heterogeneity | | | | AIC | R^2^ (conditional) |
|  |  | mode | mean | sqrt | nlvls |  |  |
| meta-analytic |  |  |  |  |  | 1977 | 0.440 |
|  |  | *I*^2^_species_ | 0.435 | 0.660 | 80 |  |  |
|  |  | *I*^2^_obs_ID_ | 0.937 | 0.968 | 594 |  |  |
| **Phylogeny** | | | | | | | |
| model |  | Heterogeneity | | | | AIC | R^2^ (conditional) |
|  |  | mode | mean | sqrt | nlvls |  |  |
| meta-analytic |  |  |  |  |  | 2007 | 4.050 |
|  |  | *I*^2^_phylogeny_ | 4.054 | 2.014 | 78 |  |  |
|  |  | *I*^2^_obs_ID_ | 0.979 | 0.989 | 594 |  |  |
| **Study ID + Species** | | | | | | | |
| model |  | Heterogeneity | | | | AIC | R^2^ (conditional) |
|  |  | mode | mean | sqrt | nlvls |  |  |
| meta-analytic |  |  |  |  |  | 1955 | 0.590 |
|  |  | *I*^2^_study_ID_ | 0.593 | 0.770 | 60 |  |  |
|  |  | *I*^2^_species_ | 0.000 | 0.001 | 80 |  |  |
|  |  | *I*^2^_obs_ID_ | 0.868 | 0.932 | 594 |  |  |
| **Study ID + Phylogeny** | | | | | | | |
| model |  | Heterogeneity | | | | AIC | R^2^ (conditional) |
|  |  | mode | mean | sqrt | nlvls |  |  |
| meta-analytic |  |  |  |  |  | 1955 | 0.600 |
|  |  | *I*^2^_study_ID_ | 0.587 | 0.766 | 60 |  |  |
|  |  | *I*^2^_phylogeny_ | 0.009 | 0.092 | 78 |  |  |
|  |  | *I*^2^_obs_ID_ | 0.867 | 0.931 | 594 |  |  |
| **Study ID + Species + Phylogeny** | | | | | | | |
| model |  | Heterogeneity | | | | AIC | R^2^ (conditional) |
|  |  | mode | mean | sqrt | nlvls |  |  |
| meta-analytic |  |  |  |  |  | 1957 | 0.590 |
|  |  | *I*^2^_study_ID_ | 0.593 | 0.770 | 60 |  |  |
|  |  | *I*^2^_species_ | 0.000 | 0.002 | 80 |  |  |
|  |  | *I*^2^_phylogeny_ | 0.000 | 0.003 | 78 |  |  |
|  |  | *I*^2^_obs_ID_ | 0.868 | 0.932 | 594 |  |  |

| **Table S4: Meta-regression results for the effects of publication year on mean and variance differences (Hedges' g as measurement).** | | | | | | | |
| --- | --- | --- | --- | --- | --- | --- | --- |
|  |  |  |  |  |  |  |  |
| **Measure** | **Method** | **Slope** | **Effects of publication year (hedges' g)** | | | **Sample sizes** | |
|  |  |  | Mean est | Lower CI | *Upper CI* | *N studies* | *k effect sizes* |
| *Hedges’ g* | *rma.mv* | Publication year | 0.172 | -0.064 | 0.409 | 59 | 594 |
|  |  |  | 0.002 | -0.024 | 0.028 |  |  |

| **Table S5: Effects of autumn seasonality versus herbivory on PSM production.** | | | | | | | | |
| --- | --- | --- | --- | --- | --- | --- | --- | --- |
|  |  |  |  |  |  |  | |  |
| **Herbivory** | | | | | | | | |
| **PSM subgroup** | **Type of estimate** | **Effect of herbivory on PSM (hedges' g)** | | | | **Sample sizes** | | |
|  |  | Mean est | Lower CI | Upper CI | pval | N studies | k effect sizes | |
| all | mean | 0.345 | -0.026 | 0.715 | 0.069 | 33 | | 345 |
| all | contrast with autumn seasonality | -0.453 | -0.916 | 0.011 | 0.056 |  | |  |
| **Autumn seasonality** | | | | | | | | |
| **PSM subgroup** | **Type of estimate** | **Effect of autumn seasonality on PSM (hedges' g)** | | | | **Sample sizes** | | |
|  |  | Mean est | Lower CI | Upper CI | pval | N studies | | k effect sizes |
| all | mean | -0.108 | -0.513 | 0.296 | 0.600 | 27 | | 249 |

| **Table S6: Effects of autumn seasonality versus herbivory on phenolic PSM subgroups (excluding other subgroups).** | | | | | | | | |
| --- | --- | --- | --- | --- | --- | --- | --- | --- |
|  |  |  |  |  |  |  | |  |
| **Herbivory** | | | | | | | | |
| **PSM group** | **Type of estimate** | **Effect of herbivory on PSM (hedges' g)** | | | | **Sample sizes** | | |
|  |  | Mean est | Lower CI | Upper CI | pval | N studies | k effect sizes | |
| phenolic | mean | 0.362 | -0.010 | 0.733 | 0.057 | 23 | | 223 |
| phenolic | contrast with autumn seasonality | -0.410 | -0.936 | 0.116 | 0.127 |  | |  |
| **Autumn seasonality** | | | | | | | | |
| **PSM group** | **Type of estimate** | **Effect of autumn seasonality on PSM (hedges' g)** | | | | **Sample sizes** | | |
|  |  | Mean est | Lower CI | Upper CI | pval | N studies | | k effect sizes |
| phenolic | mean | -0.048 | -0.421 | 0.324 | 0.799 | 23 | | 230 |

| **Table S7: Effects of herbivore class on typical PSM concentration.** | | | | | | | |
| --- | --- | --- | --- | --- | --- | --- | --- |
|  |  |  |  |  |  |  |  |
| **Clitellata** | | | | | | | |
| **PSM subgroup** | **Type of estimate** | **Effect on PSM (hedges' g)** | | | | **Sample sizes** | |
|  |  | Mean est | Lower CI | Upper CI | pval | N studies | k effect sizes |
| all | mean | -0.426 | -2.029 | 1.177 | 0.602 | 1 | 13 |
| all | contrast with Gastropoda | 1.652 | -0.733 | 4.037 | 0.175 |  |  |
| all | contrast with Insecta | 0.854 | -0.720 | 2.427 | 0.288 |  |  |
| all | contrast with Mammalia | -0.409 | -2.270 | 1.452 | 0.666 |  |  |
| all | contrast with Multiple classes | 0.351 | -1.449 | 2.150 | 0.703 |  |  |
| all | contrast with Secernentea | 1.583 | -0.501 | 3.666 | 0.137 |  |  |
| **Gastropoda** | | | | | | | |
| **PSM subgroup** | **Type of estimate** | **Effect on PSM (hedges' g)** | | | | **Sample sizes** | |
|  |  | Mean est | Lower CI | Upper CI | pval | N studies | k effect sizes |
| all | mean | 1.226 | -0.612 | 3.063 | 0.191 | 1 | 3 |
| all | contrast with Insecta | -0.799 | -2.617 | 1.020 | 0.390 |  |  |
| all | contrast with Mammalia | -2.061 | -4.181 | 0.058 | 0.057 |  |  |
| all | contrast with Multiple classes | -1.301 | -3.321 | 0.719 | 0.207 |  |  |
| all | contrast with Secernentea | -0.070 | -2.320 | 2.181 | 0.952 |  |  |
| **Insecta** | | | | | | | |
| **PSM subgroup** | **Type of estimate** | **Effect on PSM (hedges' g)** | | | | **Sample sizes** | |
|  |  | Mean est | Lower CI | Upper CI | pval | N studies | k effect sizes |
| all | mean | 0.427 | -0.092 | 0.946 | 0.107 | 27 | 300 |
| all | contrast with Mammalia | -1.263 | -2.411 | -0.114 | 0.031 |  |  |
| all | contrast with Multiple classes | -0.503 | -1.470 | 0.464 | 0.308 |  |  |
| all | contrast with Secernentea | 0.729 | -0.675 | 2.133 | 0.309 |  |  |
| **Mammalia** | | | | | | | |
| **PSM subgroup** | **Type of estimate** | **Effect on PSM (hedges' g)** | | | | **Sample sizes** | |
|  |  | Mean est | Lower CI | Upper CI | pval | N studies | k effect sizes |
| all | mean | -0.836 | -2.022 | 0.350 | 0.167 | 2 | 16 |
| all | contrast with Multiple classes | 0.760 | -0.685 | 2.204 | 0.303 |  |  |
| all | contrast with Secernentea | 1.992 | 0.210 | 3.773 | 0.028 |  |  |
| **Multiple classes** | | | | | | | |
| **PSM subgroup** | **Type of estimate** | **Effect on PSM (hedges' g)** | | | | **Sample sizes** | |
|  |  | Mean est | Lower CI | Upper CI | pval | N studies | k effect sizes |
| all | mean | -0.076 | -1.103 | 0.951 | 0.885 | 3 | 11 |
| all | contrast with Secernentea | 1.232 | -0.173 | 2.636 | 0.086 |  |  |
| **Secernentea** | | | | | | | |
| **PSM subgroup** | **Type of estimate** | **Effect on PSM (hedges' g)** | | | | **Sample sizes** | |
|  |  | Mean est | Lower CI | Upper CI | pval | N studies | k effect sizes |
| all | mean | 1.156 | -0.298 | 2.610 | 0.119 | 1 | 2 |

| **Table S8: Effects of insect order on typical PSM concentration.** | | | | | | | |
| --- | --- | --- | --- | --- | --- | --- | --- |
|  |  |  |  |  |  |  |  |
| **Coleoptera** | | | | | | | |
| **PSM subgroup** | **Type of estimate** | **Effect on PSM (hedges' g)** | | | | **Sample sizes** | |
|  |  | Mean est | Lower CI | Upper CI | pval | N studies | k effect sizes |
| all | mean | 1.034 | -0.064 | 2.132 | 0.065 | 2 | 8 |
| all | contrast with Diptera | -0.500 | -2.038 | 1.038 | 0.524 |  |  |
| all | contrast with Hemiptera | -0.934 | -2.042 | 0.174 | 0.099 |  |  |
| all | contrast with Hymenoptera | -0.961 | -2.831 | 0.910 | 0.314 |  |  |
| all | contrast with Lepidoptera | -0.657 | -1.638 | 0.324 | 0.189 |  |  |
| all | contrast with Trombidiformes | -0.142 | -1.571 | 1.287 | 0.845 |  |  |
| **Diptera** | | | | | | | |
| **PSM subgroup** | **Type of estimate** | **Effect on PSM (hedges' g)** | | | | **Sample sizes** | |
|  |  | Mean est | Lower CI | Upper CI | pval | N studies | k effect sizes |
| all | mean | 0.534 | -0.738 | 1.805 | 0.411 | 2 | 9 |
| all | contrast with Hemiptera | -0.434 | -1.756 | 0.888 | 0.520 |  |  |
| all | contrast with Hymenoptera | -0.460 | -2.478 | 1.557 | 0.655 |  |  |
| all | contrast with Lepidoptera | -0.157 | -1.394 | 1.081 | 0.804 |  |  |
| all | contrast with Trombidiformes | 0.358 | -1.244 | 1.960 | 0.662 |  |  |
| **Hemiptera** | | | | | | | |
| **PSM subgroup** | **Type of estimate** | **Effect on PSM (hedges' g)** | | | | **Sample sizes** | |
|  |  | Mean est | Lower CI | Upper CI | pval | N studies | k effect sizes |
| all | mean | 0.100 | -0.665 | 0.865 | 0.798 | 4 | 56 |
| all | contrast with Hymenoptera | -0.027 | -1.731 | 1.678 | 0.976 |  |  |
| all | contrast with Lepidoptera | 0.277 | -0.280 | 0.835 | 0.330 |  |  |
| all | contrast with Trombidiformes | 0.792 | -0.151 | 1.734 | 0.100 |  |  |
| **Hymenoptera** | | | | | | | |
| **PSM subgroup** | **Type of estimate** | **Effect on PSM (hedges' g)** | | | | **Sample sizes** | |
|  |  | Mean est | Lower CI | Upper CI | pval | N studies | k effect sizes |
| all | mean | 0.073 | -1.614 | 1.761 | 0.932 | 1 | 24 |
| all | contrast with Lepidoptera | 0.304 | -1.340 | 1.947 | 0.717 |  |  |
| all | contrast with Trombidiformes | 0.818 | -1.101 | 2.737 | 0.403 |  |  |
| **Lepidoptera** | | | | | | | |
| **PSM subgroup** | **Type of estimate** | **Effect on PSM (hedges' g)** | | | | **Sample sizes** | |
|  |  | Mean est | Lower CI | Upper CI | pval | N studies | k effect sizes |
| all | mean | 0.377 | -0.233 | 0.986 | 0.226 | 19 | 193 |
| all | contrast with Trombidiformes | 0.515 | -0.552 | 1.582 | 0.345 |  |  |
| **Trombidiformes** | | | | | | | |
| **PSM subgroup** | **Type of estimate** | **Effect on PSM (hedges' g)** | | | | **Sample sizes** | |
|  |  | Mean est | Lower CI | Upper CI | pval | N studies | k effect sizes |
| all | mean | 0.892 | -0.291 | 2.074 | 0.139 | 1 | 3 |

| **Table S9: Effects of herbivory and autumn seasonality on typical PSM production, according to plant type (woody versus herbaceous).** | | | | | | | |
| --- | --- | --- | --- | --- | --- | --- | --- |
|  |  |  |  |  |  |  |  |
| **Herbivory** | | | | | | | |
| **Plant type** | **Type of estimate** | **Effect of herbivory on PSM (hedges' g)** | | | | **Sample sizes** | |
|  |  | Mean est | Lower CI | Upper CI | pval | N studies | k effect sizes |
| herbaceous | mean | 0.683 | 0.193 | 1.174 | 0.006 | 15 | 170 |
|  | contrast with woody | -0.680 | -1.265 | -0.095 | 0.023 |  |  |
| woody | mean | 0.003 | -0.465 | 0.471 | 0.990 | 18 | 175 |
| **Autumn seasonality** | | | | | | | |
| **Plant type** | **Type of estimate** | **Effect of autumn seasonality on PSM (hedges' g)** | | | | **Sample sizes** | |
|  |  | Mean est | Lower CI | Upper CI | pval | N studies | k effect sizes |
| herbaceous | mean | -0.842 | -1.716 | 0.033 | 0.059 | 4 | 50 |
|  | contrast with woody | 0.832 | -0.072 | 1.736 | 0.071 |  |  |
| woody | mean | -0.010 | -0.439 | 0.419 | 0.965 | 23 | 199 |

| **Table S10: Effects of herbivore hours on typical PSM production.** | | | | | | | |
| --- | --- | --- | --- | --- | --- | --- | --- |
|  |  |  |  |  |  |  |  |
| **24+** | | | | | | | |
| **PSM subgroup** | **Type of estimate** | **Effect of herbivory on PSM (hedges' g)** | | | | **Sample sizes** | |
|  |  | Mean est | Lower CI | Upper CI | pval | N studies | k effect sizes |
| all | mean | 1.024 | 0.261 | 1.786 | 0.009 | 6 | 23 |
| all | contrast with 48+ | -0.504 | -0.947 | -0.061 | 0.026 |  |  |
| all | contrast with 72+ | -0.602 | -1.266 | 0.062 | 0.076 |  |  |
| all | contrast with 120+ | -0.584 | -1.365 | 0.196 | 0.142 |  |  |
| all | contrast with 200+ | -0.876 | -1.436 | -0.316 | 0.002 |  |  |
| **48+** | | | | | | | |
| **PSM subgroup** | **Type of estimate** | **Effect of herbivory on PSM (hedges' g)** | | | | **Sample sizes** | |
|  |  | Mean est | Lower CI | Upper CI | pval | N studies | k effect sizes |
| all | mean | 0.520 | -0.186 | 1.225 | 0.149 | 8 | 65 |
| all | contrast with 72+ | -0.098 | -0.737 | 0.541 | 0.764 |  |  |
| all | contrast with 120+ | -0.081 | -0.830 | 0.668 | 0.833 |  |  |
| all | contrast with 200+ | -0.372 | -0.748 | 0.004 | 0.053 |  |  |
| **72+** | | | | | | | |
| **PSM subgroup** | **Type of estimate** | **Effect of herbivory on PSM (hedges' g)** | | | | **Sample sizes** | |
|  |  | Mean est | Lower CI | Upper CI | pval | N studies | k effect sizes |
| all | mean | 0.422 | -0.372 | 1.216 | 0.298 | 6 | 73 |
| all | contrast with 120+ | 0.017 | -0.738 | 0.772 | 0.965 |  |  |
| all | contrast with 200+ | -0.274 | -0.971 | 0.423 | 0.441 |  |  |
| **120+** | | | | | | | |
| **PSM subgroup** | **Type of estimate** | **Effect of herbivory on PSM (hedges' g)** | | | | **Sample sizes** | |
|  |  | Mean est | Lower CI | Upper CI | pval | N studies | k effect sizes |
| all | mean | 0.439 | -0.395 | 1.272 | 0.302 | 7 | 63 |
| all | contrast with 200+ | -0.291 | -1.083 | 0.501 | 0.471 |  |  |
| **200+** | | | | | | | |
| **PSM subgroup** | **Type of estimate** | **Effect of herbivory on PSM (hedges' g)** | | | | **Sample sizes** | |
|  |  | Mean est | Lower CI | Upper CI | pval | N studies | k effect sizes |
| all | mean | 0.148 | -0.584 | 0.880 | 0.693 | 6 | 59 |

| **Table S11: Effects of herbivory and autumn seasonality on PSM concentrations, according to phenolic PSM subgroups. Positive Hedges' g represents an increase in PSM concentration following herbivory.** | | | | | | | |
| --- | --- | --- | --- | --- | --- | --- | --- |
|  |  |  |  |  |  |  |  |
| **Herbivory** | | | | | | | |
| **PSM subgroup** | **Type of estimate** | **Effect of herbivory (hedges' g)** | | | | **Sample sizes** | |
|  |  | Mean est | Lower CI | Upper CI | pval | N studies | k effect sizes |
| condensed tannin | mean | 0.083 | -0.586 | 0.751 | 0.808 | 4 | 25 |
|  | contrast with flavan-3-ol | 0.011 | -0.893 | 0.916 | 0.981 |  |  |
|  | contrast with flavonol | 0.611 | -0.051 | 1.272 | 0.070 |  |  |
|  | contrast with hydrolysable tannin | 0.603 | -0.305 | 1.510 | 0.193 |  |  |
|  | contrast with hydroxybenzoic acid | 0.050 | -0.742 | 0.842 | 0.902 |  |  |
|  |  |  |  |  |  |  |  |
| flavan-3-ol | mean | 0.094 | -0.771 | 0.958 | 0.831 | 3 | 11 |
|  | contrast with flavonol | 0.600 | -0.257 | 1.456 | 0.170 |  |  |
|  | contrast with hydrolysable tannin | 0.591 | -0.453 | 1.636 | 0.267 |  |  |
|  | contrast with hydroxybenzoic acid | 0.039 | -0.968 | 1.045 | 0.940 |  |  |
|  |  |  |  |  |  |  |  |
| flavonol | mean | 0.693 | 0.185 | 1.202 | 0.008 | 9 | 50 |
|  | contrast with hydrolysable tannin | -0.008 | -0.761 | 0.744 | 0.983 |  |  |
|  | contrast with hydroxybenzoic acid | -0.561 | -1.243 | 0.121 | 0.107 |  |  |
|  |  |  |  |  |  |  |  |
| hydrolysable tannin | mean | 0.685 | -0.057 | 1.428 | 0.070 | 3 | 24 |
|  | contrast with hydroxybenzoic acid | -0.553 | -1.480 | 0.374 | 0.243 |  |  |
|  |  |  |  |  |  |  |  |
| hydroxybenzoic acid | mean | 0.132 | -0.512 | 0.776 | 0.687 | 5 | 34 |
| **Autumn seasonality** | | | | | | | |
| **PSM subgroup** | **Type of estimate** | **Effect of autumn seasonality (hedges' g)** | | | | **Sample sizes** | |
|  |  | Mean est | Lower CI | Upper CI | pval | N studies | k effect sizes |
| condensed tannin | mean | 1.110 | 0.148 | 2.071 | 0.024 | 5 | 8 |
|  | contrast with flavan-3-ol | -0.255 | -1.384 | 0.873 | 0.658 |  |  |
|  | contrast with flavonol | -1.188 | -2.225 | -0.151 | 0.025 |  |  |
|  | contrast with hydrolysable tannin | -1.341 | -2.417 | -0.264 | 0.015 |  |  |
|  | contrast with hydroxybenzoic acid | -1.612 | -2.691 | -0.533 | 0.003 |  |  |
|  |  |  |  |  |  |  |  |
| flavan-3-ol | mean | 0.855 | 0.131 | 1.578 | 0.021 | 7 | 19 |
|  | contrast with flavonol | -0.933 | -1.704 | -0.161 | 0.018 |  |  |
|  | contrast with hydrolysable tannin | -1.086 | -1.972 | -0.199 | 0.016 |  |  |
|  | contrast with hydroxybenzoic acid | -1.357 | -2.204 | -0.509 | 0.002 |  |  |
|  |  |  |  |  |  |  |  |
| flavonol | mean | -0.078 | -0.586 | 0.429 | 0.762 | 10 | 74 |
|  | contrast with hydrolysable tannin | -0.153 | -0.820 | 0.515 | 0.654 |  |  |
|  | contrast with hydroxybenzoic acid | -0.424 | -1.166 | 0.318 | 0.263 |  |  |
|  |  |  |  |  |  |  |  |
| hydrolysable tannin | mean | -0.231 | -0.805 | 0.343 | 0.430 | 6 | 68 |
|  | contrast with hydroxybenzoic acid | -0.271 | -1.093 | 0.550 | 0.518 |  |  |
|  |  |  |  |  |  |  |  |
| hydroxybenzoic acid | mean | -0.502 | -1.166 | 0.162 | 0.138 | 8 | 24 |

| **Table S12: Effects of precipitation and temperature on PSM concentration, according to phenolic PSM subgroup. Positive Hedges' g represents an increase in PSM concentration following precipitation or temperature.** | | | | | | | |
| --- | --- | --- | --- | --- | --- | --- | --- |
|  |  |  |  |  |  |  |  |
| **Precipitation** | | | | | | | |
| **PSM subgroup** | **Type of estimate** | **Effect of precipitation on PSM (hedges' g)** | | | | **Sample sizes** | |
|  |  | Mean est | Lower CI | Upper CI | pval | N studies | k effect sizes |
| condensed tannin | mean | 3.541 | -1.132 | 8.213 | 0.138 | 1 | 2 |
|  | contrast with flavan-3-ol | -2.728 | -7.441 | 1.984 | 0.257 |  |  |
|  | contrast with flavonol | -3.562 | -8.252 | 1.128 | 0.137 |  |  |
|  | contrast with hydrolysable tannin | -4.135 | -8.842 | 0.573 | 0.085 |  |  |
|  | contrast with hydroxybenzoic acid | -4.327 | -9.023 | 0.370 | 0.071 |  |  |
|  |  |  |  |  |  |  |  |
| flavan-3-ol | mean | 0.812 | -0.016 | 1.640 | 0.054 | 7 | 19 |
|  | contrast with flavonol | -0.834 | -1.753 | 0.086 | 0.076 |  |  |
|  | contrast with hydrolysable tannin | -1.406 | -2.466 | -0.346 | 0.009 |  |  |
|  | contrast with hydroxybenzoic acid | -1.598 | -2.668 | -0.529 | 0.003 |  |  |
|  |  |  |  |  |  |  |  |
| flavonol | mean | -0.021 | -0.543 | 0.501 | 0.937 | 10 | 74 |
|  | contrast with hydrolysable tannin | -0.573 | -1.411 | 0.265 | 0.180 |  |  |
|  | contrast with hydroxybenzoic acid | -0.765 | -1.681 | 0.151 | 0.102 |  |  |
|  |  |  |  |  |  |  |  |
| hydrolysable tannin | mean | -0.594 | -1.268 | 0.080 | 0.084 | 6 | 53 |
|  | contrast with hydroxybenzoic acid | -0.192 | -1.210 | 0.826 | 0.712 |  |  |
|  |  |  |  |  |  |  |  |
| hydroxybenzoic acid | mean | -0.786 | -1.587 | 0.015 | 0.055 | 7 | 23 |
| **Temperature** | | | | | | | |
| **PSM subgroup** | **Type of estimate** | **Effect of temperature on PSM (hedges' g)** | | | | **Sample sizes** | |
|  |  | Mean est | Lower CI | Upper CI | pval | N studies | k effect sizes |
| condensed tannin | mean | 2.309 | -0.269 | 4.887 | 0.079 | 4 | 6 |
|  | contrast with flavan-3-ol | -0.573 | -3.497 | 2.350 | 0.701 |  |  |
|  | contrast with flavonol | -2.001 | -4.730 | 0.729 | 0.151 |  |  |
|  | contrast with hydrolysable tannin | -1.816 | -4.684 | 1.053 | 0.215 |  |  |
|  | contrast with hydroxybenzoic acid | -2.943 | -5.349 | -0.537 | 0.017 |  |  |
|  |  |  |  |  |  |  |  |
| flavan-3-ol | mean | 1.736 | 0.322 | 3.149 | 0.016 | 6 | 18 |
|  | contrast with flavonol | -1.427 | -3.062 | 0.208 | 0.087 |  |  |
|  | contrast with hydrolysable tannin | -1.243 | -3.117 | 0.632 | 0.194 |  |  |
|  | contrast with hydroxybenzoic acid | -2.370 | -4.295 | -0.444 | 0.016 |  |  |
|  |  |  |  |  |  |  |  |
| flavonol | mean | 0.309 | -0.621 | 1.238 | 0.515 | 9 | 73 |
|  | contrast with hydrolysable tannin | 0.185 | -1.058 | 1.427 | 0.771 |  |  |
|  | contrast with hydroxybenzoic acid | -0.943 | -2.564 | 0.679 | 0.255 |  |  |
|  |  |  |  |  |  |  |  |
| hydrolysable tannin | mean | 0.493 | -0.782 | 1.768 | 0.448 | 4 | 45 |
|  | contrast with hydroxybenzoic acid | -1.127 | -2.969 | 0.715 | 0.231 |  |  |
|  |  |  |  |  |  |  |  |
| hydroxybenzoic acid | mean | -0.634 | -1.970 | 0.702 | 0.352 | 6 | 21 |

**Table S13: List of authors used for data extraction in meta-analysis.**

| **Author** | **Year** | **Study ID** | **Study type** |
| --- | --- | --- | --- |
| Ahmed, Z.B. et al. | 2017 | s4 | seasonality |
| Anderson, P. and Agrell, J. | 2005 | h26 | herbivory |
| Banuelos, M. et al. | 2004 | h29 | herbivory |
| Berezina, E.V. et al. | 2017 | s15 | seasonality |
| Bernal, M. et al. | 2013 | s9 | seasonality |
| Bixenmann, R.J. et al. | 2016 | h2 | herbivory |
| Blanchard, M. and Bowers, M.D. | 2020 | s14 | seasonality |
| Boeckler, G.A. et al. | 2013 | h30 | herbivory |
| Calf, O.N. et al. | 2020 | h13 | herbivory |
| Cappellari, L.R. et al. | 2020 | h10 | herbivory |
| Covelo, F. et al. | 2001 | s11 | seasonality |
| Eisenring, M. et al. | 2018 | h18 | herbivory |
| Forbes, T.D.A. et al. | 1995 | s13 | seasonality |
| Gera Hol, W.H. et al. | 2004 | h27 | herbivory |
| Gomez, J.D. et al. | 2018 | h9 | herbivory |
| Gori, A. et al. | 2020 | s2 | seasonality |
| Gregianini, T.S. et al. | 2004 | s22 | seasonality |
| Gutbrodt, B. et al. | 2011 | h24 | herbivory |
| Hall, C. et al. | 2018 | h8 | herbivory |
| Ito, M. et al. | 2017 | s16 | seasonality |
| Kinahan, I.G. et al. | 2020 | s23 | seasonality |
| Kos, M. et al. | 2012 | h23 | herbivory |
| Kunkler, N. et al. | 2013 | h32 | herbivory |
| Liu, M. et al. | 2018 | h7 | herbivory |
| Liu, Y. et al. | 2016 | s5 | seasonality |
| Liu, Z. et al. | 1998 | s12 | seasonality |
| Liu, Z. et al. | 2010 | h25 | herbivory |
| Martin, N. and Muller, C. | 2007 | h15 | herbivory |
| Masa, C.V. et al. | 2016 | s6 | seasonality |
| Mitra, S. et al. | 2019 | h11 | herbivory |
| Mundim, F.M. et al. | 2021 | h16 | herbivory |
| Nybakken, L. et al. | 2013 | s10 | seasonality |
| Papazian, S. et al. | 2019 | h14 | herbivory |
| Pascual-Alvarado, E. et al. | 2008 | h33 | herbivory |
| Prasannalaxmi, K. and Rani, U. | 2016 | h21 | herbivory |
| Qianqian, S. et al. | 2018 | s18 | seasonality |
| Rea, R.V. and Gillingham, M.P. | 2001 | s21 | seasonality |
| Rea, R.V. and Gillingham, M.P. | 2001 | h31 | herbivory |
| Salminen, J-P. et al. | 2004 | s25 | seasonality |
| Scholz, S.S. et al. | 2014 | h6 | herbivory |
| Scogings, P.F. et al. | 2014 | h1 | herbivory |
| Sirvent, T.M., Krasnoff, S.B., Gibson, D.M. | 2003 | h28 | herbivory |
| Soltanabad, M.H. et al. | 2018 | s3 | seasonality |
| Steinbauer, M.J. et al. | 2015 | s8 | seasonality |
| Thakur, M. et al. | 2020 | h12 | herbivory |
| Tuominen, A. and Salminen, J-P. | 2017 | s27 | seasonality |
| Vijaya, M. and Rani, P.U. | 2017 | h20 | herbivory |
| Visakorpi, K. et al. | 2020 | h4 | herbivory |
| Wang, D. et al. | 2020 | h17 | herbivory |
| War, A.R. et al. | 2013 | h22 | herbivory |
| Wigley, B. et al. | 2019 | h5 | herbivory |
| Williams, R.S. And Howells, J.M. | 2018 | h19 | herbivory |
| Yao, I. et al. | 2018 | s17 | seasonality |
| Yarnes, C.T. et al. | 2008 | s24 | seasonality |
| Zebelo, S. et al. | 2017 | h3 | herbivory |
| Zhang, L. et al. | 2016 | s7 | seasonality |
| Zhang, L. et al. | 2020 | s19 | seasonality |
| Zhu, J. et al. | 2020 | s20 | seasonality |
| Zlatic, N. et al. | 2019 | s1 | seasonality |
|  |  |  |  |

REFERENCE:

Bañuelos, M.-J., Sierra, M., & Obeso, J.-R. (2004). Sex, secondary compounds and asymmetry. Effects on plant–herbivore interaction in a dioecious shrub. Acta Oecologica, 25, 151 – 157.

Ben Ahmed, Z., Yousfi, M., Viaene, J., Dejaegher, B., Demeyer, K., Mangelings, D., & Vander Heyden, Y. (2017). Seasonal, gender and regional variations in total phenolic, flavonoid, and condensed tannins contents and in antioxidant properties from Pistacia atlantica ssp. leaves. Pharmaceutical Biology, 55, 1185 – 1194.

Berezina, E. V., Brilkina, A. A., & Veselov, A. P. (2017). Content of phenolic compounds, ascorbic acid, and photosynthetic pigments in Vaccinium macrocarpon Ait. Dependent on seasonal plant development stages and age (the example of introduction in Russia). Scientia Horticulturae, 218, 139 – 146.

Bernal, M., Llorens, L., Julkunen-Tiitto, R., Badosa, J., & Verdaguer, D. (2013). Altitudinal and seasonal changes of phenolic compounds in Buxus sempervirens leaves and cuticles. Plant Physiology and Biochemistry, 70, 471 – 482.

Bixenmann, R. J., Coley, P. D., Weinhold, A., & Kursar, T. A. (2016). High herbivore pressure favors constitutive over induced defense. Ecology and Evolution, 6, 6037 – 6049.

Blanchard, M., & Bowers, M. D. (2020). Critical phenological events affect chemical defense of plant tissues: Iridoid glycosides in a woody shrub. Journal of Chemical Ecology, 46, 206 – 216.

Calf, O. W., Lortzing, T., Weinhold, A., Poeschl, Y., Peters, J. L., Huber, H., Steppuhn, A., & van Dam, N. M. (2020). Slug feeding triggers dynamic metabolomic and transcriptomic responses leading to induced resistance in Solanum dulcamara. Frontiers in Plant Science, 11, 803.

Covelo, F., & Gallardo, A. (2001). Temporal variation in total leaf phenolics concentration of Quercus robur in forested and harvested stands in northwestern Spain. Canadian Journal of Botany, 79, 1262 – 1269.

Dai, X., Han, M., Liu, Q., Shang, G., Yin, B., Wang, A., Dean, B. E., Wei, W., & Yang, S. (2014). Seasonal changes in the concentrations of plant secondary metabolites and their effects on food selection by Microtus oeconomus. Mammalian Biology, 79, 215 –220.

Del Rosario Cappellari, L., Chiappero, J., Palermo, T. B., Giordano, W., & Banchio, E. (2020). Impact of soil rhizobacteria inoculation and leaf-chewing insect herbivory on Mentha piperita leaf secondary metabolites. Journal of Chemical Ecology, 46, 619 – 630.

Eisenring, M., Glauser, G., Meissle, M., & Romeis, J. (2018). Differential impact of herbivores from three feeding guilds on systemic secondary metabolite induction, phytohormone levels and plant-mediated herbivore interactions. Journal of Chemical Ecology, 44, 1178 – 1189.

Forbes, T. D. A., Pemberton, I. J., Smith, G. R., & Hensarling, C. M. (1995). Seasonal variation of two phenolic amines in Acacia berlandieri. Journal of Arid Environments, 30, 403 – 415.

Gera Hol, W. H., Macel, M., van Veen, J. A., & van der Meijden, E. (2004). Root damage and aboveground herbivory change concentration and composition of pyrrolizidine alkaloids of Senecio jacobaea. Basic and Applied Ecology, 5, 253 – 260.

Gori, A., Nascimento, L. B., Ferrini, F., Centritto, M., & Brunetti, C. (2020). Seasonal and diurnal variation in leaf phenolics of three medicinal mediterranean wild species: What is the best harvesting moment to obtain the richest and the most antioxidant extracts? Molecules, 25, 956.

Gregianini, T. S., Porto, D. D., Nascimento, N. C. D., Fett, J. P., Henriques, A. T., & Fett-Neto, A. G. (2004). Environmental and ontogenetic control of accumulation of brachycerine, a bioactive indole alkaloid from Psychotria brachyceras. Journal of Chemical Ecology, 30, 2023 – 2036.

Hall, C. R., Robertson, L. P., Carroll, A. R., & Kitching, R. L. (2018). The effect of Psephodiplosis rubi (Diptera: Cecidomyiidae) leaf galls on the secondary metabolite profiles of two congeneric host plants. Austral Entomology, 57, 228 – 237.

Ito, M., Tamura, N., & Hayashi, F. (2017). Seasonal changes in leaf chemistry and leaf selection of the Japanese giant flying squirrel upon two tree species. Ecology and Evolution, 7, 5766 – 5773.

Kinahan, I. G., Rigsby, C. M., Savage, S. K., Houseman, N. L., Marsella, A. S., Oppong-Quaicoe, A., DeBoef, B. L., Orians, C. M., & Preisser, E. L. (2020). Seasonal changes in eastern hemlock (Tsuga canadensis) foliar chemistry. Canadian Journal of Forest Research, 50, 557 – 564.

Kos, M., Houshyani, B., Wietsma, R., Kabouw, P., Vet, L. E. M., van Loon, J. J. A., & Dicke, M. (2012). Effects of glucosinolates on a generalist and specialist leaf-chewing herbivore and an associated parasitoid. Phytochemistry, 77, 162 – 170.

Künkler, N., Brandl, R., & Brändle, M. (2013). Changes in clonal poplar leaf chemistry caused by stem galls alter herbivory and leaf litter decomposition. PLoS One, 8, e79994.

Liu, M., Zhou, F., Pan, X., Zhang, Z., Traw, M. B., & Li, B. (2018). Specificity of herbivore-induced responses in an invasive species, Alternanthera philoxeroides (alligator weed). Ecology and Evolution, 8, 59 – 70.

Liu, Y., Qian, C., Ding, S., Shang, X., Yang, W., & Fang, S. (2016). Effect of light regime and provenance on leaf characteristics, growth and flavonoid accumulation in Cyclocarya paliurus (Batal) Iljinskaja coppices. Botanical Studies, 57, 28.

Liu, Z., Cai, Y., Fang, Y., Jing, J., & Li, K. (2010). Induced response in Schima superba : Effects of earlyseason herbivory on leaf traits and subsequent insect attack. African Journal of Biotechnology, 9, 8731 – 8738.

Liu, Z., Carpenter, S. B., Bourgeois, W. J., Yu, Y., Constantin, R. J., Falcon, M. J., & Adams, J. C. (1998). Variations in the secondary metabolite camptothecin in relation to tissue age and season in Camptotheca acuminata. Tree Physiology, 18, 265 – 270.

Mitra, S., Mobarak, S. H., Karmakar, A., & Barik, A. (2019). Activities of antioxidant enzymes in three species of Ludwigia weeds on feeding by Altica cyanea. Journal of King Saud University-Science, 31, 1522 – 1527.

Mundim, F. M., Vieira-Neto, E. H. M., Alborn, H., & Bruna, E. M. (2021). Disentangling the influence of water limitation and simultaneous above and belowground herbivory on plant tolerance and resistance to stress. Journal of Ecology, 109, 2729 – 2739.

Nybakken, L., Selås, V., & Ohlson, M. (2013). Increased growth and phenolic compounds in bilberry (Vaccinium myrtillus L.) following forest clear-cutting. Scandinavian Journal of Forest Research, 28, 319 – 330.

Papazian, S., Girdwood, T., Wessels, B. A., Poelman, E. H., Dicke, M., Moritz, T., & Albrectsen, B. R. (2019). Leaf metabolic signatures induced by real and simulated herbivory in black mustard (Brassica nigra). Metabolomics, 15, 130.

Pascual-Alvarado, E., Cuevas-Reyes, P., Quesada, M., & Oyama, K. (2008). Interactions between galling insects and leaf-feeding insects: The role of plant phenolic compounds and their possible interference with herbivores. Journal of Tropical Ecology, 24, 329 – 336.

Prasannalaxmi, K., & Rani, U. (2016). Interactions between herbivore Leucinodes orbonalis G. and its host plant Solanum melongena L.: A study on insect induced direct plant responses. Allelopathy Journal, 37, 273 – 286.

Qianqian, S., Xianying, F., Haijun, Z., Zunling, Z., & Linguo, Z. (2018). Seasonal changes in the major flavonoids and anti-tumor capacity of the methanol extracts of carpinus. Current Topics in Nutraceutical Research, 16, 179 – 187.

Rea, R. V., & Gillingham, M. P. (2001). The impact of the timing of brush management on the nutritional value of woody browse for moose Alces alces. Journal of Applied Ecology, 38, 710 – 719.

Salminen, J.-P., Roslin, T., Karonen, M., Sinkkonen, J., Pihlaja, K., & Pulkkinen, P. (2004). Seasonal variation in the content of hydrolyzable tannins, flavonoid glycosides, and proanthocyanidins in oak leaves. Journal of Chemical Ecology, 30, 1693 – 1711.

Scholz, S. S., Vadassery, J., Heyer, M., Reichelt, M., Bender, K. W., Snedden, W. A., Boland, W., & Mithöfer, A. (2014). Mutation of the Arabidopsis calmodulin-like protein CML37 deregulates the Jasmonate pathway and enhances susceptibility to herbivory. Molecular Plant, 7, 1712 – 1726.

Scogings, P. F., Hjältén, J., Skarpe, C., Hattas, D., Zobolo, A., Dziba, L., & Rooke, T. (2014). Nutrient and secondary metabolite concentrations in a savanna are independently affected by large herbivores and shoot growth rate. Plant Ecology, 215, 73 – 82.

Sirvent, T. M., Krasnoff, S. B., & Gibson, D. M. (2003). Induction of hypericins and hyperforins in Hypericum perforatum in response to damage by herbivores. Journal of Chemical Ecology, 29, 2667 – 2681.

Soltanabad, M. H., Bagherieh-Najjar, M. B., & Mianabadi, M. (2018). Seasonal variations in carnosic acid content of rosemary correlates with anthocyanins and soluble sugars. Journal of Medicinal Plants and By-product, 7, 163 – 171.

Steinbauer, M. J., Wallis, I. R., Davies, N. W., & Watson, S. J. (2015). Foliar quality of co-occurring mallee eucalypts: Balance of primary and secondary metabolites reflects past growing conditions. Chemoecology, 25, 179 – 191.

Thakur, M. P., Künne, T., Unsicker, S. B., Biere, A., Ferlian, O., Pruschitzki, U., Thouvenot, L., Türke, M., & Eisenhauer, N.(2021). Invasive earthworms reduce chemical defense and increase herbivory and pathogen infection in native trees. Journal of Ecology, 109, 763 – 775.

Tuominen, A., & Salminen, J.-P. (2017). Hydrolyzable tannins, flavonol glycosides, and phenolic acids show seasonal and ontogenic variation in Geranium sylvaticum. Journal of Agricultural and Food Chemistry, 65, 6387 – 6403.

Valares Masa, C., Sosa Díaz, T., Alías Gallego, J. C., & Chaves Lobón, N. (2016). Quantitative variation of flavonoids and diterpenes in leaves and stems of Cistus ladanifer L. at different ages. Molecules, 21, 275.

Vijaya, M., & Rani, P. U. (2017). Defensive responses in Capsicum annuum (L) plants, induced due to the feeding by different larval instars of Spodoptera litura (F). Arthropod-Plant Interactions, 11, 193 – 202.

Visakorpi, K., Riutta, T., Malhi, Y., Salminen, J.-P., Salinas, N., & Gripenberg, S. (2020). Changes in oak (Quercus robur) photosynthesis after winter moth (Operophtera brumata) herbivory are not explained by changes in chemical or structural leaf traits. PLoS One, 15, e0228157.

Wang, D., Wang, Q., Sun, X., Gao, Y., & Ding, J. (2020). Potato tuberworm Phthorimaea operculella (Zeller) (Lepidoptera: Gelechioidea) leaf infestation affects performance of conspecific larvae on harvested tubers by inducing chemical defenses. Insects, 11, 633.

War, A. R., Paulraj, M. G., Ignacimuthu, S., & Sharma, H. C. (2013). Defensive responses in groundnut against chewing and sapsucking insects. Journal of Plant Growth Regulation, 32, 259 – 272.

Wigley, B. J., Coetsee, C., Augustine, D. J., Ratnam, J., Hattas, D., & Sankaran, M. (2019). A thorny issue: Woody plant defence and growth in an east African savanna. Journal of Ecology, 107, 1839 – 1851.

Williams, R. S., & Howells, J. M. (2018). Effects of intraspecific genetic variation and prior herbivory in an old-field plant on the abundance of the specialist aphid Uroleucon nigrotuberculatum (Hemiptera: Aphididae). Environmental Entomology, 47, 422 – 431.

Yao, I. (2019). Seasonal changes in the density of the symbionts Buchnera and Wolbachia of the aphid Tuberculatus macrotuberculatus on Quercus dentata. Entomologia Experimentalis et Applicata, 167, 261 – 268.

Yarnes, C. T., Boecklen, W. J., & Salminen, J.-P. (2008). No simple sum: Seasonal variation in tannin phenotypes and leaf-miners in hybrid oaks. Chemoecology, 18, 39 – 51.

Zebelo, S., Disi, J., Balusu, R., Reeves, B., & Fadamiro, H. (2017). Spodoptera exigua modulates gossypol biosynthesis in cotton Gossypium hirsutum. Journal of Plant Interactions, 12, 121 – 127.

Zhang, L., Yang, M., Gao, J., Jin, S., Wu, Z., Wu, L., & Zhang, X. (2016). Seasonal variation and gender pattern of phenolic and flavonoid contents in Pistacia chinensis Bunge inflorescences and leaves. Journal of Plant Physiology, 191, 36 – 44.

Zhang, L., Zhang, S., Ye, G., & Qin, X. (2020). Seasonal variation and ecological importance of tannin and nutrient concentrations in Casuarina equisetifolia branchlets and fine roots. Journal of Forestry Research, 31, 1499 – 1508.

Zhu, J., Xu, Q., Zhao, S., Xia, X., Yan, X., An, Y., Mi, X., Guo, L., Samarina, L., & Wei, C. (2020). Comprehensive coexpression analysis provides novel insights into temporal variation of flavonoids in fresh leaves of the tea plant (Camellia sinensis). Plant Science, 290, 110306.

Zlatić, N., Jakovljević, D., & Stanković, M. (2019). Temporal, plant part, and interpopulation variability of secondary metabolites and antioxidant activity of Inula helenium L. Plants, 8, 179.
